# Effect of UVC Irradiation on the Oxidation of Histidine in Monoclonal Antibodies

**DOI:** 10.1038/s41598-020-63078-5

**Published:** 2020-04-14

**Authors:** Yuya Miyahara, Koya Shintani, Kayoko Hayashihara-Kakuhou, Takehiro Zukawa, Yukihiro Morita, Takashi Nakazawa, Takuya Yoshida, Tadayasu Ohkubo, Susumu Uchiyama

**Affiliations:** 1 Graduate School of Pharmaceutical Sciences, Osaka University, Osaka, Japan; 2 Graduate School of Engineering, Osaka University, Osaka, Japan; 3 Panasonic Co., Ltd., Osaka, Japan; 4 Department of Chemistry, Nara Women’s University, Nara, Japan

**Keywords:** Chemical modification, Proteins, Mass spectrometry

## Abstract

We oxidized histidine residues in monoclonal antibody drugs of immunoglobulin gamma 1 (IgG1) using ultraviolet C irradiation (UVC: 200–280 nm), which is known to be potent for sterilization or disinfection. Among the reaction products, we identified asparagine and aspartic acid by mass spectrometry. In the photo-induced oxidation of histidine in angiotensin II, ^18^O atoms from H_2_^18^O in the solvent were incorporated only into aspartic acid but not into asparagine. This suggests that UVC irradiation generates singlet oxygen and induces [2 + 2] cycloaddition to form a dioxetane involving the imidazole C^γ^ − C^δ2^ bond of histidine, followed by ring-opening in the manner of further photo-induced retro [2 + 2] cycloaddition. This yields an equilibrium mixture of two keto-imines, which can be the precursors to aspartic acid and asparagine. The photo-oxidation appears to occur preferentially for histidine residues with lower p*K*_a_ values in IgG1. We thus conclude that the damage due to UVC photo-oxidation of histidine residues can be avoided in acidic conditions where the imidazole ring is protonated.

## Introduction

Therapeutic antibodies are widely used in the treatment of diseases such as cancers and immunological disorders. Because the degradation of protein-based drugs during storage is inevitable, it is necessary to establish methods to characterize the degradation process and to assess the safety of the drugs^1^. One of the most significant causes of degradation is the oxidation of the side chain of an amino-acid residue, which often plays a key role in protein stability and function^2^. The imidazole group of histidine (His) is particularly susceptible to photo-oxidation, as are the indole ring of tryptophan (Trp) and the phenol group of tyrosine (Tyr)^3^.

Irradiation of ultraviolet A (UVA) light with the wavelength range 320–400 nm is known to cause photo-oxidation of His to yield aspartic acid (Asp) and asparagine (Asn) in the presence of a photosensitizer^4,5^. In addition, irradiation with both visible (400–700 nm) and UVA light also cause the decomposition of His residues in antibody drugs^3,6^. This suggests that irradiation with UVC light can be more harmful to antibody drugs, owing to its higher energy associated with the shorter wavelength than UVA or UVB (280–320 nm) even in the absence of a photosensitizer. In spite of such a risk of protein degradation, UVC irradiation known as a potent means of ultraviolet germicidal irradiation (UVGI) has been used for disinfection by damaging the DNA of microrganisms^7^. We thus expect that UVC irradiation can be a better substitute for the disinfection of antibody drugs than UVA or UVB, under the condition that the effects of UVC irradiation on proteins are fully understood and special care for the possible degradation is taken accordingly. However, there are only a few studies concerning the degradation of proteins by UVC irradiation. The reported modifications of amino acid residues include the oxidation of methionine residues in antibody drugs and the photolysis of disulfide bonds in serum proteins^8,9^. It is thus necessary to characterize the degradation products of UVC-irradiated proteins and to elucidate the mechanism of each reaction.

In this study, we used mass spectrometry to address the issue of UVC-induced degradation of monoclonal antibody (mAb) drugs by identifying the modification products, especially those from His residues. In the additional experiments with angiotensin II as a model peptide, Asp and Asn were identified as well as their derivative but their possible precursors such as the hydroperoxide corresponding to an oxygen adduct of His were not found to occur during photo-oxidation, unlike the case with UVA and UVB irradiations^4,5^. Based on these results that show the oxidation of His residues to be purely a photochemical process, we elaborated a possible reaction mechanism involving a photo-induced concerted reaction of the imidazole group of His and singlet oxygen.

## Results

### The photo-oxidation of antibodies by UVC irradiation

**Figure 1:**
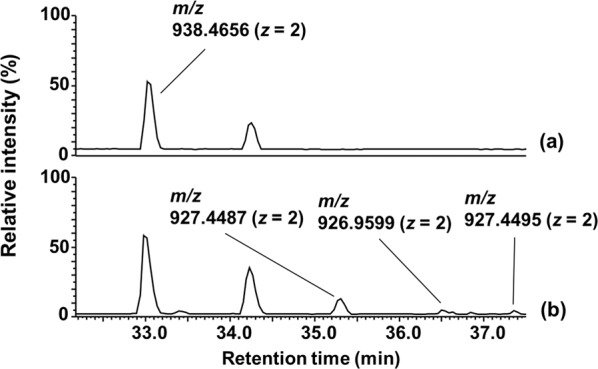
Base peak ion chromatograms of the tryptic digests of *S*-CAM-adalimumab before (**a**) and after UVC irradiation for 30 min (**b**) in the range of retention times 32 to 38 min. The peptide eluted at 34.2 min was identified as SLSLSPG with a mass of 659.35 Da corresponding to the C-terminal peptide of adalimumab heavy chain (Supplementary Fig. S4, Supplementary Table S3).

**Figure 2:**
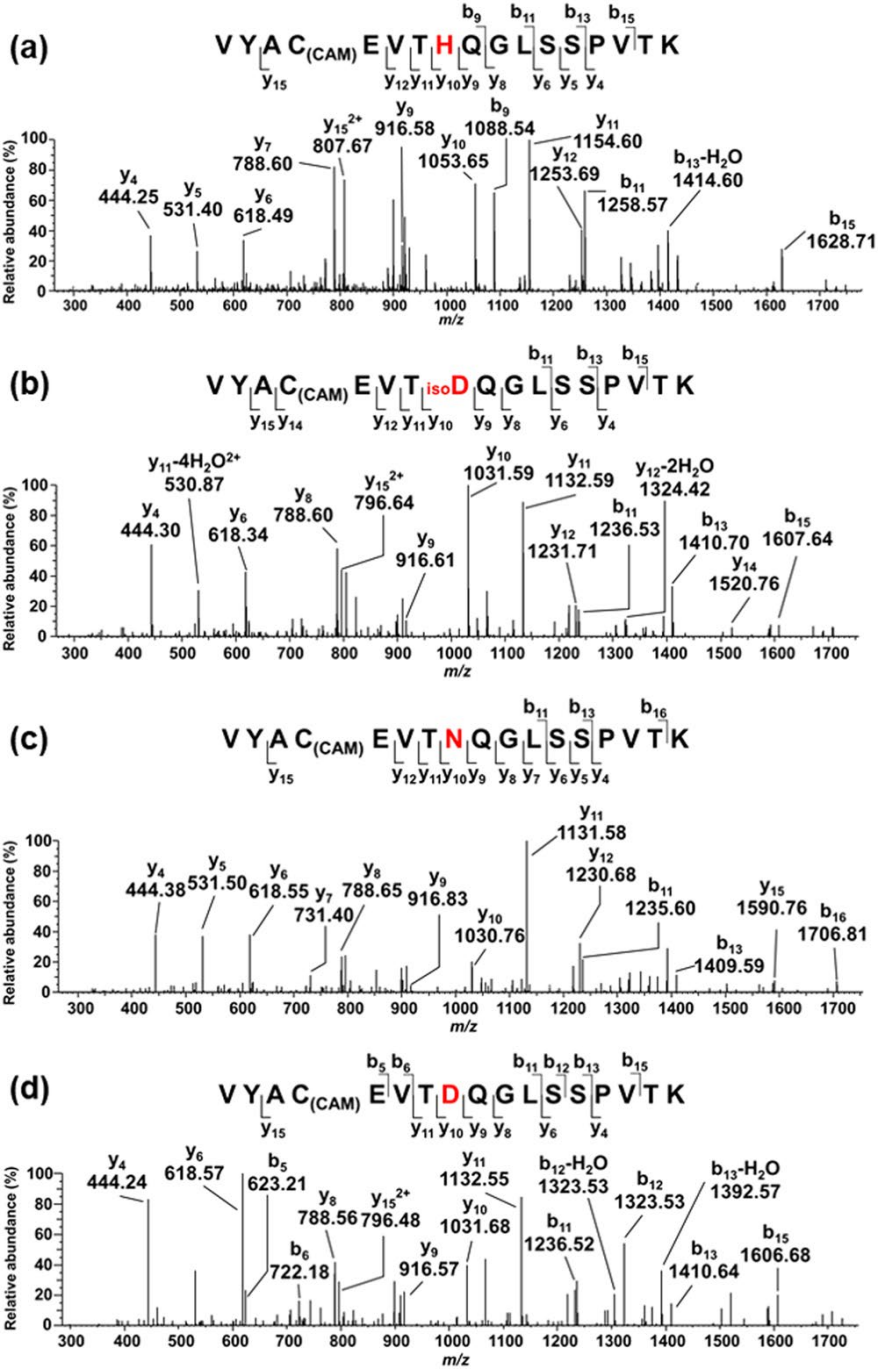
Tandem mass spectra of the peptides VYAC_(CAM)_E- VT**X**QGLSSPVTK corresponding to the residues 191–207, in which **X** = His198 (**a**) eluted at 33.0 min (Fig. 1) is modified. The *m/z* values of individual precursor ions are shown at the tops of the peaks in Fig. 1. (**b**) **X** = iso-Asp, eluted at 35.3 min, (**c**) **X** = Asn eluted at 36.3 min, and (**d**) **X** = Asp, eluted at 37.4 min.

The emission spectrum of the PDUVL has a peak at 237 nm and covers a broad range of UVC wavelengths (Supplementary Fig. S1). Following irradiation of adalimumab and rituximab with this UVC light, the modification products of individual His residues were analyzed by peptide mapping. The time course of the photo-oxidation was monitored by the measurement of base peak ion chromatograms of their tryptic digests (For the full area, see Supplementary Fig. S2). Figure 1 shows part of the chromatogram, in which the base ion peak at *m/z* 938.4656 (*z* = 2) appeared for a peptide eluted at the retention time of 33.0 min. This peptide was identified as residue 191–207 of both adalimumab and rituximab with the amino acid sequence: VYAC_(CAM)_EVTHQGLSSPVTK (calculated monoisotopic mass of 1874.92 Da; C_(CAM)_ stands for *S*-carboxamidomethylcysteine) as a result of MS/MS analysis. Irradiating this peptide with UVC light for 30 min yielded at least three peptides eluted at retention times of 35.4, 36.3, and 37.6 min (Fig. 1b). Considering the almost identical mass values of peaks appeared at *m/z* 927.4487 and 927.4495 for peptides eluted at 35.4 and 37.6 min, respectively, amounting to the loss of mass by 22 Da from the residue mass of His, there is a possibility that these peptides contain l- and d-isomers of Asp and/or those of iso-Asp as the oxidation products derived from a His residue. MS/MS analysis of their respective base-ion peaks revealed that His198 was modified by the UVC irradiation to give Asp, according to the mass difference of 115 Da between the fragment-ion peaks y_9_ (*m/z* 916.6) and y_10_ (*m/z* 1031.6) as shown in Fig. 2b,d. To distinguish between the possible products of Asp and iso-Asp, we noted the difference in their fragmentation patterns of MS/MS spectra, expecting that iso-Asp residues show the enhanced b- or y-type ion on the N-terminal side^10^. As shown in Fig. 2b, the y_10_ ion peak signifying the fragmentation at the N-terminal side of Asp is appreciably more intense than that of the y_9_ ion arising from the cleavage at the C-terminal side, while this relationship is much less pronounced in the MS/MS spectrum shown in Fig. 2d. These findings suggest that the peptides eluted at 35.4 min and 37.7 min contains iso-Asp and Asp, respectively. The peptide eluted at 36.3 min exhibited a base ion peak at *m/z* 926.9599 (*z* = 2). Comparing the closely similar MS/MS spectra shown in Figs. 2b–d, and the difference of 1 Da between the y_10_ ion peaks at *m/z* 1031.59 in Fig. 2b and at *m/z* 1030.76 in Fig. 2c, we can identify Asn unambiguously at residue 198.

Similarly, photo-oxidation affected almost all the His residues to give both Asp (and/or iso-Asp) and Asn with a variety of proportions without appreciable preference to any of these products (Supplementary Table S1). For His189, which appeared only in a peptide HKVYACEVTHQGLSSPVTK (residue189–207) as a precursor to VYACEVTHQGLSSPVTK (residue191–207), it was difficult to quantify the degradation ratio (*D*r) because none of the peaks of the product peptides were detected at intensities above the cutoff level of 0.3% compared with the maximal peak intensity of the total ion chromatogram.

Unlike the oxidation of His under the exposure to standard daylight (*i.e*., a D65 lamp) to form oxo-histidine (residue mass is 151 Da), and the possible [4 + 2]-cycloaddition products including a 1,2-dioxolane and and a γ-hydroxyhydanthoin (see Supplementary Fig. S5 for structures) with the increment of mass by 32 Da and 48 Da, respectively^6^, we did not detect any of such prospective peaks in the present experiments with a UVC lamp. This suggests that photo-oxidation induced by UVC irradiation could possibly proceed in a manner different from the conventional mechanism involving radical species generated by peroxides or UVA (and UVB) irradiation in the presence of a photosensitizer^11–14^.

### Photo-oxidation of the His residue in angiotensin II by UVC irradiation

To elucidate the mechanism of photo-oxidation of His, we exposed angiotensin II to UVC irradiation both in water (H_2_^16^O) and in hydrogen heavy oxide (H_2_^18^O) for 60 min. The peak appearing at the retention time of 8.2 min exhibited a base ion peak at *m/z* 523.7855 (*z* = 2), matched with the molecular mass of intact angiotensin II (1045.5345 Da) with the sequence DRVYIHPF. We acquired the MS/MS data from this peak as a reference for analyzing the fragment-ion peaks of the oxidation products (Supplementary Fig. S6a,b).

**Figure 3:**
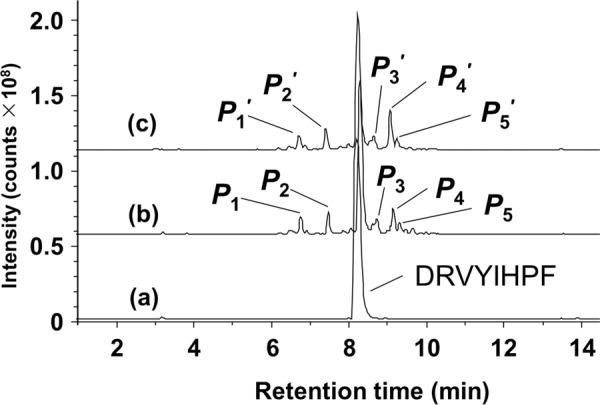
Base peak ion chromatograms of angiotensin II [DRVYIHPF; retention time 8.2 min, *m/z* 523.79 (*z *= 2)] (**a**), and its oxidation products after UVC irradiation in H_2_^16^O (**b**) and in H_2_^18^O (**c**). The retention times and *m/z* values of the peaks are *P*_1_, *P*_1_′: 6.7 min, *m/z* 379.72 (*z *= 2); *P*_2_, *P*_2_′: 7.5 min, *m/z* 263.13 (*z *= 1); *P*_3_, *P*_3_′: 8.7 min, *m/z* 501.78 (*z *= 2); *P*_4_, *P*_4_′: 9.1 min, *m/z* 512.27 (*z *= 2); and *P*_5_, *P*_5_′: 9.3 min, *m/z* 512.76 (*z *= 2, *P*_5_) and 513.76 (*z *= 2, *P*_5_′).

**Figure 4:**
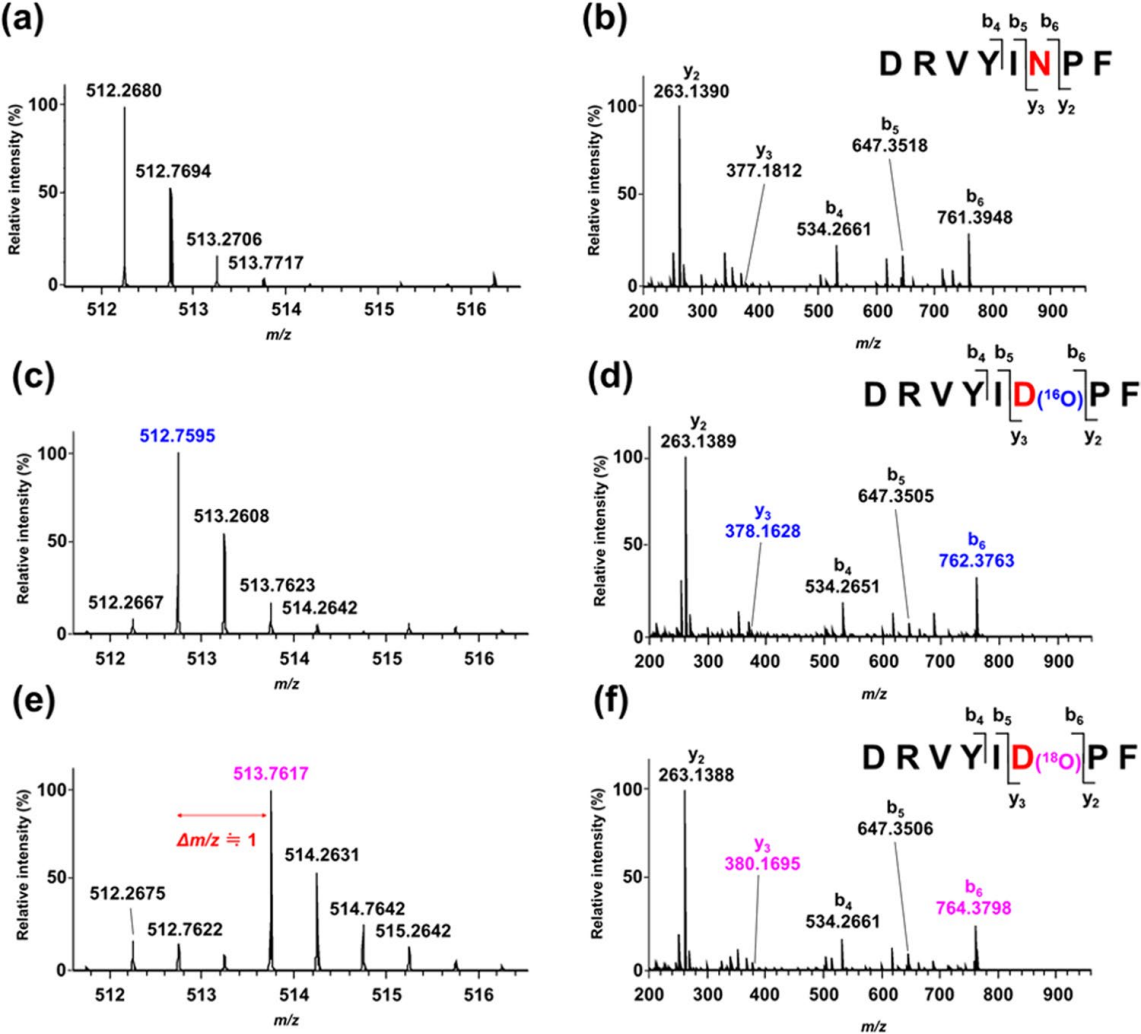
(**a**) Mass spectrum of the photo-oxidation product *P*_4_′ that appeared as a peak at 9.2 min in Fig. 3c. (**b**) MS/MS spectrum of the precursor peak at *m/z* 512.2680 in spectrum (**a**). (**c**) Mass spectrum of the photo-oxidation product *P*_5_ that appeared as a peak at 9.4 min in Fig. 3b. (d) MS/MS spectrum of the base peak at *m/z* 512.7595 in spectrum (**c**). (**e**) Mass spectrum of the photo-oxidation product *P*_5_′ that appeared as a peak at 9.4 min in Fig. 3c. (**f**) MS/MS spectrum of the base peak at *m/z* 513.7617 in (**e**). All the spectra were taken for the solutions of angiotensin II after UVC irradiation for 60 min in H_2_^16^O (**c, d**) and in H_2_^18^O (**a, b, e and f**).

As shown in Fig. 3a,b, the peptide peaks appeared at retention times of 6.7 min (*P*_1_), 7.5 min (*P*_2_), 8.7 min (*P*_3_), 9.2 min (*P*_4_), and 9.3 min (*P*_5_) in the base-ion chromatogram of the photo-oxidation products. The base-ion peaks at *m/z* 512.27 (*P*_4_) and *m/z* 512.76 (*P*_5_) are diagnostics for the peptides containing Asn (*P*_4_) and Asp (*P*_5_) (Supplementary Fig. S7a and Fig. 4c). Their respective retention times are consistent with the elution profiles of authentic peptides and are predictable by a sequence-specific retention calculator^15,16^. The masses of *P*_4_ and *P*_4_′ were identical (1022.5 Da) irrespective of the reactions in H_2_^16^O and in H_2_^18^O, indicating that heavy oxygen (^18^O) of H_2_^18^O was not incorporated into Asn (Fig. 4a,b, Supplementary Fig. S7a,b). In contrast, the mass of *P*_5_′ (1025.5 Da) obtained in H_2_^18^O was heavier by 2 Da than that of *P*_5_ (1023.5 Da) obtained in H_2_^16^O, indicating that one atom of heavy oxygen (^18^O) was incorporated into Asp (Fig. 4c,e).

Comparing the MS/MS spectra of the peaks *P*_5_ and *P*_5_′, the b_6_ ion peak at *m/z* 762.3763 and the y_3_ ion peak at *m/z* 378.1628 observed for *P*_5_ were shifted to *m/z* 764.3798 and *m/z* 380.1695 for *P*_5_′, respectively, with an increment of mass by 2 Da. In contrast, the b_5_ ion peak at *m/z* 647.3505 and y_2_ ion peak at *m/z* 263.1389 for *P*_5_ were virtually unchanged for *P*_5_′. These findings of fragment peaks observed in the MS/MS spectra of *P*_5_ and *P*_5_′ clearly show that ^18^O from H_2_^18^O is incorporated into the β-carboxyl group of Asp.

Additional peaks also appeared for the products *P*_1_ eluted at 6.7 min and *P*_2_ eluted at 7.5 min (Fig. 3). From the base-ion peak at *m/z* 379.7197 (*z* = 2) and its fragment-ion peaks (Supplementary Fig. S8), we identified *P*_1_ as DRVYIN-imide (see the structure in Supplementary Fig. S9a) with a mass of 760.39 Da. We detected the peak of product *P*_2_ at *m/z* 263.1391 ([M + H]^+^), corresponding to the mass of 262.14 Da, and identified it as a dipeptide PF (Supplementary Fig. S10). This peptide is obviously derived from the peptide *P*_4_ or *P*_5_ by the cleavage of the peptide bond at the N-terminal side of proline (P) residue to form *P*_1_. The minor peak *P*_3_ eluted at 8.7 min exhibited a base-ion peak at *m/z* 501.7804 (*z* = 2). The intensity of peak *P*_3_ was, however, not sufficient enough to obtain the MS/MS spectrum needed to characterize the chemical structure of this peptide (Supplementary Fig. S11).

### Measurement of imidazole C2-H/D exchange rates of Histidine residues in adalimumab

We determined the p*K*_a_ values of individual His residues by the mass spectrometric measurement of imidazole C2-H/D exchange rates because the difference in the susceptibility toward photo-oxidation in adalimumab and rituximab can reflect the ionic states in the solution at pH 4.95 and 6.10, respectively. The p*K*_a_ value is obtained from the measurement of H/D-exchange rate in terms of pseudo-first-order rate constant ${k}_{\varphi }$ and its maximal value of ${k}_{\varphi }^{{\rm{\max }}}$ (Supplementary Fig. S12), which is closely associated with solvent accessible surface area (*ASA*)^17^. We thus calculated the relative solvent accessibility (*RSA*), based on the X-ray crystallographic data of adalimumab and rituximab. The values of *RSA*, p*K*_a_ and *k*_2_ are summarized in Supplementary Table S2. Due to the extremely slow rate of H/D-exchange reaction, the values of p*K*_a_ and *k*_2_ were not measurable for His57, His314 and His198, while we set the incubation time to 336 h, which might be long enough to allow for the measurement of the H/D-exchange reaction with the half-life of 2–3 weeks. This is consistent with the considerably low solvent accessibility of these residues.

## Discussion

**Figure 5:**
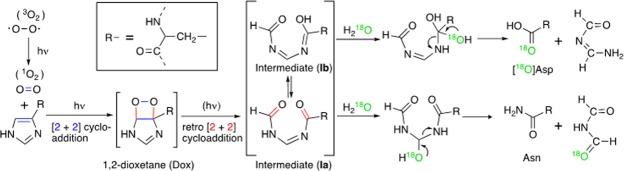
A possible reaction mechanism for UVC-induced oxidation of histidine.

The mechanism of photo-induced oxidation of His in the presence of a photosensitizer has been scrutinized for nearly half a century^4,5,11,12^. Many researchers have assumed the formation of singlet (^1^O_2_ or ^1^Δ_g_) oxygen due to irradiation with UVA or UVB, followed by the generation of hydroperoxide, probably through a radical species^13,14^. Hydroperoxides of aromatic amino acids are generally unstable and decompose further into the corresponding carbonyl and carboxylic acid derivatives. The photo-oxidation of His yields a complex mixture of products, including Asp and Asn, that would reflect multiple reaction pathways^11^. Consequently, the effect of photo-oxidation is variable and depend on reaction conditions such as the temperature and pH. Despite the high photon energies associated with wavelengths <280 nm, the UVC-induced oxidation of His produces Asp and Asn almost exclusively, but much less effectively compared with the UVA or UVB irradiation, as shown in Supplementary Table S1. This suggests that UVC irradiation specifically enhances the reactivity of oxygen with the imidazole group of His, while keeping the phenolic side chain of Tyr intact, as demonstrated in the model experiment with angiotensin II. As a plausible mechanism of photo-oxidation, we consider the possibility of photo-induced [2 + 2] cycloaddition between singlet oxygen and the C^γ^ − C^δ2^ double bond of the imidazole group to form an intermediate involving the dioxetane (Dox) ring (Fig. 5). Although the pericyclic [2 + 2] cycloaddition is symmetry-forbidden under thermal conditions, it is a typical photochemical reaction, which is allowed to proceed *via* an excited state.

According to the Woodward-Hoffmann rule, Dox in the excited state is allowed to undergo retro [2 + 2] cycloaddition (or cycloelimination)^18^, enabling the O − O and C − C bonds to break simultaneously to form a pair of carbonyl groups in the product, which also exist in excited states. Note that both the reactions of cycloaddition and retro-cycloaddition occur consecutively, requiring one photon as the sole source to initiate the reaction. The derivatives of Dox are relatively stable, despite the highly strained structure of the four-membered ring, because the [2 + 2] cycloelimination that causes their decomposition is symmetry-forbidden in the ground state. Therefore, the absence of Dox, which should have a residue mass of 169 Da (His + O_2_), suggests that this process may require Dox to be in an excited state. Although cleavage of the O − O and C − C bonds of Dox could also occur through a thermal two-step mechanism involving a biradical intermediate^18^, it is difficult to find any evidence supporting such a multistep mechanism, consisting of concerted [2 + 2] cycloaddition and subsequent thermal radical reaction, for the formation and decomposition of Dox, in preference to the simpler one-step concerted mechanism that takes advantage of robust UVC irradiation.

The cleavage of the C^γ^ − C^δ2^ bond through photo-induced retro [2 + 2] cycloaddition represents one of the most significant features of the mechanism shown in Fig. 5, which involves the intermediate I formally represented by the equilibrium mixture of tautomeric isomers Ia and Ib not only as direct products of the C^γ^ − C^δ2^ bond cleavage but also as precursors to Asp and Asn. Allowing for the incorporation of ^18^O atoms only in Asp but not in Asn during the photo-oxidation of His in the H_2_^18^O solution, the intermediate species Ia and Ib are hydrolyzed to Asp and Asn, respectively, depending on the locations of the cleavable C = N linkages in the tautomeric isomers.

The symmetry-allowed thermal [4 + 2] cycloaddition of ^1^O_2_ to His in the manner of the Diels-Alder reaction has been proposed as an alternative mechanism for oxidation, especially in the presence of a photosensitizer^2,4,5,11,19^. The suggested mechanism involves the degradation of intermediate endoperoxide into β-aspartylurea, from which Asp and Asn are derived. However, this mechanism is inconsistent with the present finding that no ^18^O atoms from H_2_^18^O were incorporated into Asn. In particular, there is no reaction mechanism reliable enough to explain the requisite cleavage of the C^γ^ − C^δ2^ bond (Supplementary Fig. S5).

While the present reaction mechanism based on [2 + 2] cycloaddition and elimination could rationalize the formation of Asp and Asn in the photo-oxidation of His198 (Fig. 2), it was necessary to assume an additional cyclic succinimide intermediate to interpret the conversion of intermediate I to iso-Asp (Supplementary Fig. S9a). Note that this cyclic intermediate can be the precursor not only to iso-Asp but also to Asp, depending on the cleavage of one of the C-N bonds in the succinimide ring. Such a cyclic structure has not been identified in the photo-oxidation products of mAbs but found in the form of DRVYIN-imide (*P*_1_ in Fig. 3) occurring in the photo-oxidation product of angiotensin II. It is possible to consider intermediate Ia as a precursor to the succinimide formed through the attack of the imide C = N nitrogen on the peptidyl α-carbonyl carbon atom, followed by the cleavage of one of the C-N bond to form the imide of DRVYIN with the release of the C-terminal dipeptide of Pro-Phe detected in the peak *P*_2_ (Supplementary Fig. S9a). This mechanism also implies the involvement of racemization to occur in the succinimide ring, resulting in d-Asp as another photo-oxidation product of His (Supplementary Fig. S9b)^20^. However, it is likely that the peptide containing d-Asp, if any, is coeluted with that containing l-Asp, because the sole difference in the chirality of Asp198 in a 17-amino-acid-long peptide could not affect a significant change in the LC retention time (Fig. 1b).

**Figure 6:**
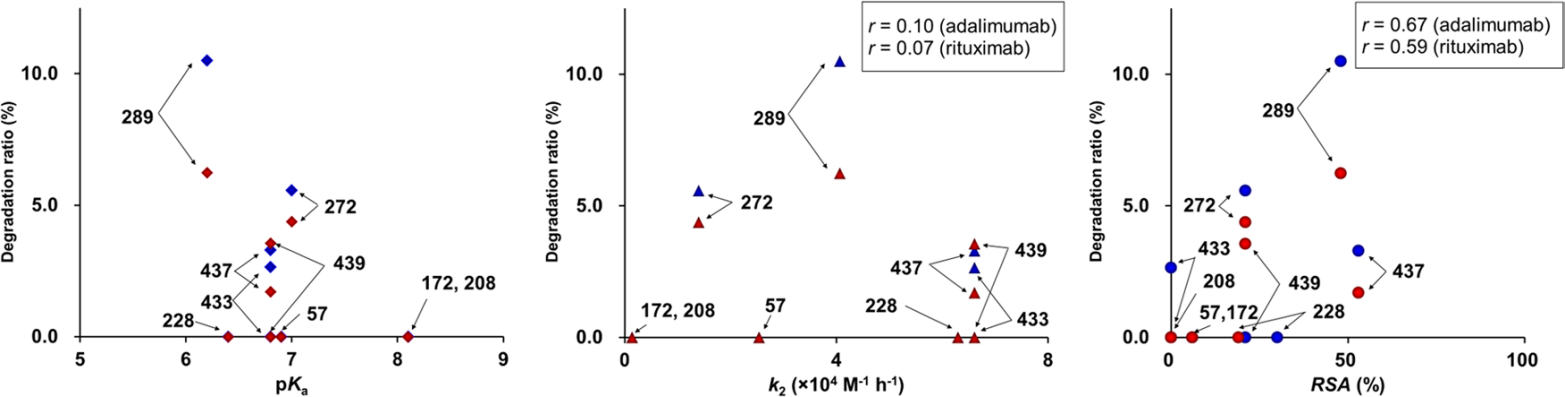
The relationships between the degradation ratio of photo-oxidation products of His in mAb and the quantities p*K*_a_ (left), *k*_2_ (middle), and *RSA* (right), for adalimumab at pH 4.95 (red) and rituximab at 6.10 (blue). We calculated the correlation coefficients *r* between the degradation ratio and parameters *k*_2_ and *RSA*.

The reaction mechanism of [2 + 2] cycloaddition for the photo-oxidation of the His residue in mAb drugs predicts that singlet oxygen reacts preferentially with the electrically neutral imidazole group rather than its cationic form. This is because the protonation of the imidazole group may compromise the proper double-bond character of the C^γ^ − C^δ2^ bond to react with the singlet oxygen. In fact, it can be clearly discerned that the susceptibility of a His residue to photo-oxidation depends on its p*K*_a_ value (Fig. 6). Moreover, His residues were photo-oxidized invariably higher in the rituximab solution at pH 6.10 than at pH 4.95 for adalimumab. A typical example is His289 with p*K*_a_ 6.2, which indicates that His289 is almost fully protonated in adalimumab at pH 4.95, while only about half of it is protonated in rituximab at pH 6.10. This enhances the degradation ratio (*D*r), which we defined as a measure of efficiency of photo-oxidation, from 6.2% at pH 4.95 to 10.5% at pH 6.10 (Supplementary Table S2). Note that the calculated *RSA* of this residue is 48% for both of the mAb drugs, making solvent accessibility less likely to be the main factor influencing the reactivity of His to singlet oxygen under UVC irradiation.

We also compared the susceptibility of each His residue (*D*r) with the second-order rate constant *k*_2_ of the H/D exchange reaction as another index of photo-oxidation, expecting that both of these parameters can have a positive correlation with the accessibility of each His residue to solvent^21^. Fig. 6 clearly shows that the susceptibility of each His residue to photo-oxidation depends strongly on the p*K*_a_ value as well as *RSA*, because His439 with the highest values of H/D-exchange rate and *RSA* is considerably less sensitive to photo-oxidation and His289 appeared to be most sensitive to oxidation is most acidic of all (Fig. 6). From these results, it is possible to conclude that the photo-oxidation of His residues occur preferably in their non-protonated forms as revealed by the higher reactivity of His residues with low p*K*_a_ values, signifying their ease of deprotonation in weakly acidic to neutral solutions. This is in accord with the reaction mechanism assuming the [2 + 2] cycloaddition of singlet oxygen to the C^γ^ − C^δ2^ double bond of the imidazole ring in its non-protonated form.

Many previous reports suggest that the conversion of His affects the function of antibodies. His268 (His 272 in this study) is associated with the function of complement-dependent cytotoxicity activity and antibody-dependent cell-mediated cytotoxicity activity^22,23^. Moore *et al*. reported that the Fc mutations S267E/H268F/S324T enhanced C1q binding in IgG1^24^. Mimoto *et al*. demonstrated that the Fc mutant including H268D increased affinity to FcγR (Fc gamma receptor) IIIa^25^. Additionally, the His310 (His314 in this study), His433 (His437 in this study), and His435 (His439 in this study) in the CH2-CH3 hinge play important roles in the FcRn-Fc interaction of IgG1^26–28^. Our results also showed that the His residues located in these Fc regions were damaged with UVC irradiation. The functional significance of these His residues indicates the importance of developing an effective protocol to avoid their damage by UVC irradiation, which has a prospect of being used wider as a potent means for sterilization or disinfection of mAb drugs. One of the main features of the reaction mechanism shown in Fig. 5 is that it does not involve radical species responsible for causing oxidative modifications of Trp, Tyr, His, and Met residues. ^18^O atoms from H_2_^18^O in the solvent were incorporated only into Asp oxidized from His in adalimumab (Supplementary Fig. S13). Because the photo-oxidation by UVC irradiation occurs preferably for His residues with lower p*K*_a_ values, it is possible to suppress the photo-oxidation by lowering the pH of the solution so that the imidazole group of His is protonated. In this study, we focused on histidine, which is most susceptible to singlet oxygen^29^. Considering that Trp is also susceptible to the [2 + 2] cycloaddition of singlet oxygen at the C^γ^ − C^δ1^ double bond of the indole ring, we are now extending the search for the products occurred by the UVC irradiation of adalimumab and rituximab to identify the possible modification products of Trp as well as those corresponding to the cleavage products of angiotensin II. The results of further studies concerning the effects of UVC irradiation on Trp, Tyr, and other oxidation-sensitive amino acids will be reported elsewhere. The comprehensive knowledge concerning the effects of potent UVC light on proteins should be indispensable for the safe use of UVC irradiation for degrading viral DNA or RNA, while minimizing the damage of proteins.

## Methods

### Materials

We purchased the therapeutic mAb immunoglobulin (IgG) gamma 1 drugs adalimumab and rituximab from Eisai Co., Ltd. (Tokyo, Japan) and Chugai Pharmaceutical Co., Ltd. (Tokyo, Japan), respectively. In addition, we purchased Tris-HCl buffer (pH 8.0), 10% trifluoroacetic acid, 0.1% formic acid, acetonitrile 0.1% formic acid and 7 K Dialysis Casettes from Thermo Fisher Scientific (San Jose, CA, USA). We obtained 8 M guanidine hydrochloride from Sigma-Aldrich (St. Louis, MO), dithiothreitol, iodoacetamide, sodium acetate, 2-morpholinoethanesulfonic acid, 4-(2-hydroxyethyl)-1-piperazinepropanesulfonic acid and deuterium oxide from FUJIFILM Wako Pure Chemical Co., Ltd. (Tokyo, Japan), and MicroSpin G-25 columns from GE Healthcare (Chicago, IL). We purchased trypsin from Promega Co. (Madison, WI); angiotensin II from Peptide Institute, Inc. (Osaka, Japan); and ^18^O-water, acetonitrile (LC-MS grade), and ordinary water (LC-MS grade) from Merck (Darmstadt, HE).

### Sample preparation and UVC irradiation

We prepared all the solutions of the mAb drugs to be subjected to UVC irradiation at the concentration of 5 mg/mL in water or in hydrogen heavy oxide (H_2_^18^O). The pH values of the aqueous solutions of adalimumab and rituximab were 4.95 and 6.10, respectively. We deliberately avoided using buffer solutions to exclude the possibility of interference due to anions and cations other than H^+^ and OH^−^. We used a Planar Deep Ultraviolet Light Source (PDUVL: Panasonic Co., Ltd., Osaka, Japan) as the UVC light source. We performed all the irradiation experiments in a 0.1 cm quartz cuvette light path. We irradiated the solutions of the mAb drugs with the PDUVL for a variety of time periods up to 30 min (0, 1, 5, 15, and 30 min.) at room temperature. We fixed the UVC illuminance at 1.26 × 10^3^ J/cm^2^, as measured using a KI/KIO_3_ chemical actinometer^30^, by adjusting the distance between the sample solution and the light source appropriately.

### Peptide mapping

We reduced the mAb proteins (20 μg) with and without UVC irradiation in 8 M guanidine hydrochloride (66 μL) with dithiothreitol (3 μL) at 37 °C for 30 min, and alkylated the newly formed thiol group of cysteine by adding 100 mM of iodoacetamide (7 μL/Milli-Q water) at room temperature in the dark for 15 min. After quenching the alkylation by adding 4 μL of dithiothreitol, we exchanged the buffers of the samples with 100 mM Tris−HCl buffer (pH 8.0) using the MicroSpin G-25 columns. The reduced and alkylated protein was digested with trypsin (2 μg) and incubated at 37 °C for 12 hours. The digestion was stopped by adding 10 μL of 10% trifluoroacetic acid. The digested samples were separated by reversed-phase chromatography using an Ultimate 3000 system (Thermo Fisher Scientific) employing an ACQUITY UPLC Peptide BEH C18, 130 Å, 1.7 μm, 2.1 mm × 150 mm (Waters, Milford MA) column, heated at 50 °C. Mobile phase A was water/0.1% formic acid, and mobile phase B was acetonitrile/0.1% formic acid. The flow rate was 0.2 mL/min, and we carried out a linear gradient program for mobile phase B (from 3% to 40%, 80 min). We detected the eluent with a mass spectrometer, LTQ/XL Orbitrap (Thermo Fisher Scientific, Waltham, MA), equipped with an electrospray ion source in the positive-ion mode for the *m/z* range from 150 to 2000. For MS/MS fragmentation analysis, the parent ions were fragmented using collision-induced dissociation at an isolation width of 4 Da and a collision energy of 35 V. We employed BioPharmaFinder software (Thermo Fisher Scientific) to map the mAb sequence and identify the modification sites. To identify peptides containing His and the other photo-sensitive residues, we applied a mass change within the defined range from -58 to +162 Da, taking particular care of the increment of mass by 32 Da (+ O_2_), 48 Da (+O_3_), 4 Da (β-ureidoAsp), and all the other presumable products appeared in the mechanism shown in Fig. 5 and Supplementary Figs. S5 and S9. We followed the default settings of automatic peak search program for the identification of peaks with BioPharmaFinder. For example, the level of enzyme specificity was set to “Strict” and the filter function of mass accuracy to less than 10 ppm for the *m/z* values of MS peaks^31^.

### Relative solvent accessibility and pKa values of His residues in mAb drugs

We generated the full-length IgG1 model structure using the crystal structure of human IgG (PDB ID: 1HZH). We calculated the relative solvent accessibility (*RSA*) of the side chains for the His residue from the ratio of the solvent accessible surface area (*ASA*) to the maximum possible solvent accessible surface area (*ASA*^max^):

$$RSA=100\cdot ASA/AS{A}^{max},$$

where *ASA* allows for the surrounding amino-acid residues and *ASA*^max^ corresponds to the *ASA* value for the residue fully exposed to solvent. We calculated these values with Molecular Operating Environment version 2018 (MOLSYS Inc., Tokyo, Japan). Because of the asymmetrical nature of the three-dimensional structures of the mAb molecules, we took the mean values of *RSA* for the heavy and light chains.

The p*K*_a_ value and the second-order rate constant *k*_2_ of the hydrogen/deuterium (H/D) exchange reaction at the imidazole C2 (histidine C^ε1^) position of each His residue were measured by the mass spectrometric titration of pseudo-first-order rate constant ${k}_{\varphi }$ of the H/D-change reaction against pH as reported in our previous publications^6,17,21^. The D_2_O solutions of buffers used were 50 mM sodium acetate (pH 3.5–4.5), 50 mM MES (pH 5.0–7.0), and 50 mM HEPES (pH 7.5–9.5). The protein sample (5 mg/mL) in each one of these solutions was incubated at 25 °C for the H/D exchange reaction. After incubation for a defined time, the protein sample was processed in the same manner as peptide mapping as described above. The time-dependent change of isotopic pattern was monitored for each tryptic peptide containing a His residue, of which *k*_φ_ value is given by the equation:^17^

$${k}_{\varphi }=\,\mathrm{ln}(1+{R}_{t}-{R}_{0})/t,$$

where *R*_*t*_ is the intensity ratio *I*_*M*_(*t*)/*I*_*M*+1_(*t*) of the isotopic peaks corresponding to the monoisotopic masses *M* and (*M+*1) at an incubation time *t* (h) and *R*_0_ is the value of *R*_*t*_ at *t* = 0, thereby we took *t* = 336 h in the present study. The p*K*_a_ and ${k}_{\varphi }^{{\rm{\max }}}$ values were obtained from the inflection points and the upper asymptote of the titration curve of ${k}_{\varphi }$ versus pH, respectively, by using a JMP software (SAS Institute Inc., Cary, NC, USA) implementing the 4-parameter logistic fitting. The rate constant *k*_2_ is represented by the equation:

$${k}_{2}={k}_{\varphi }^{{\rm{\max }}}({K}_{{\rm{a}}}/{K}_{{\rm{W}}}),$$

which involves these experimental parameters and the ion-product of water *K*_W_ = 1 × 10^−14^ mol^2^ L^−2 ^^17,21^.

### UVC irradiation of angiotensin II and LC/MS analysis

We dissolved angiotensin II to a concentration of 2 mg/mL in water (H_2_^16^O) or in hydrogen heavy oxide (H_2_^18^O). The pH of the solution was 6.29. We performed all the irradiation experiments in a 0.1 cm quartz cuvette light path. We irradiated the angiotensin II solutions for 0 and 60 min with UVC emitted from the PDUVL at room temperature. After irradiation, we submitted a five-fold diluted solution (5 μL) to an LC20 system (Shimadzu, Kyoto, Japan) with an ACQUITY UPLC Peptide BEH C18 column (130 Å, 1.7 μm, 2.1 mm × 150 mm) at a column temperature of 50 °C. We carried out the reversed-phase liquid chromatography (LC) separation as described above, with a slight modification of the gradient of the mobile phase B (from 2% to 80%, 13 min).

The eluates were introduced directly into an ESI-MS system consisting of a Q-TOF mass spectrometer, maXis II™ ETD (Bruker Daltonics, Billerica, MA). We calibrated the mass values by using ESI-L Low-Concentration Tuning Mix (Agilent Technologies, Palo Alto, CA). All the measurements were performed in the positive-ion mode. We analyzed the peptide fragments using data-analysis software (Bruker Daltonics).

### The efficiency of photo-oxidation

To compare the susceptibility of His residues to photo-oxidation, we calculated the degradation ratio (*D*r) defined by the relative peak area of the respective peptides in an extracted ion chromatogram as follows:

$$Dr({\rm{ \% }})=100(\frac{{A}_{H}}{{A}_{H}+{A}_{X}\,}),$$

where *A*_H_ is the peak area of a peptide containing an unreacted His residue, and *A*_X_ (X = Asp or Asn) is that of peptide in which His residue is oxidized to either Asp or Asn. We include the peak areas of peptides containing both Asp and iso-Asp in *A*_D_. We set a cutoff level of 0.3% of the maximal peak intensity in each total ion chromatogram to obtain *D*r, even if a peak detected below the cutoff leval could be identified as a product of photo-oxidation.

## Supplementary information

Supplementary information
