# Supplementary material for: Effect of UVC Irradiation on the Oxidation of Histidine in Monoclonal Antibodies
Source: Sci Rep. 2020 Apr 14;10:6333. doi: 10.1038/s41598-020-63078-5 (PMC7156388; doi:10.1038/s41598-020-63078-5)
Supplement: Supplementary file 1 — Supplementary information [file 41598_2020_63078_MOESM1_ESM.docx]

Supplementary Information

Effect of UVC Irradiation on the Oxidation of Histidine in Monoclonal Antibodies

Yuya Miyahara^1^, Koya Shintani^2^, Kayoko Kakuhou Hayashihara^2^, Takehiro Zukawa^3^, Yukihiro Morita^3^, Takashi Nakazawa^4^, Takuya Yoshida^1^, Tadayasu Ohkubo^1,*^, Susumu Uchiyama^2,*^^*^

^1^ Graduate School of Pharmaceutical Sciences, Osaka University

^2^ Graduate School of Engineering, Osaka University

^3^ Panasonic Co., Ltd.

^4^ Department of Chemistry, Nara Women’s University

**Contents**

Supplementary Figure S1. Emission spectrum of the UVC light source PDUVL. 2

Supplementary Figure S2. Base peak ion chromatograms of peptides in the tryptic digests of adalimumab (a) initial and adalimumab after UVC irradiation for (b) 1 min, (c) 5 min, (d) 15 min, and (e) 30min. 3

Supplementary Figure S3. Mass spectrum of the peptide that appeared as a peak at 33.0 min (Fig. 1a). 4

Supplementary Figure S4. (a) Mass spectrum of the peptide that appeared as a peak at 34.2 min (Fig. 1a). (b) MS/MS spectrum of the precursor peak at *m/z* 660.3560 in (a). 5

Supplementary Figure S5. (a) A mechanism of sensitized photo-oxidation of His as proposed by Tomita *et al*. (b) The synthesis and reaction of 5-alkyl-5-hydroxy-hydantoin, which has been isolated as a stable compound and characterized 6

Supplementary Figure S6. (a) Mass spectrum of the peptide that appeared as a peak at 8.3 min (Fig. 3a). (b) MS/MS spectrum of the precursor peak at *m/z* 523.7855 in (a). 7

Supplementary Figure S7. (a) Mass spectrum of the photo-oxidation product *P*_4_ that appeared as a peak at 9.2 min (Fig. 3b). (B) MS/MS spectrum of the precursor peak at *m/z* 512.2673 in spectrum (a). 8

Supplementary Figure S8. (a) Mass spectrum of the photo-oxidation product *P*_1_ that appeared as a peak at 6.7 min (Fig. 3B). (b) MS/MS spectrum of the precursor peak at *m/z* 379.7197 in (a). Enlargements of the regions *m/z* 386 – 390 (c) and *m/z* 485 – 495 (d) in spectrum (b). 9

Supplementary Figure S9a. A possible mechanism for the cleavage of the Asn-Pro bond to form the C-terminal Asn-imide. 10

Supplementary Figure S9b. An extended mechanism for the formation of oxidation products including the racemic mixture of Asp residues. 11

Supplementary Figure S10. Mass spectrum of the photo-oxidation product *P*_2_ that appeared as a peak at 7.5 min (Fig. 3b). 12

Supplementary Figure S11. Mass spectrum of the photo-oxidation product *P*_3_ that appeared as a minor peak at 8.8 min (Fig. 3b). 13

Supplementary Figure S12. pH dependence of pseudo-first-order rate constants for H/ D exchange at the C2-position of the histidine residues 14

Supplementary Figure S13. Mass spectrum of the peptide was identified as residue 279-292 of adalimumab containing His289 in the amino acid se-quence of FNWYVDGVEV(H oxidized to D)NAK 15

Supplementary Table S1. Degradation ratio of individual histidine residues in mAb drugs after UVC irradiation for 30 min. 16

Supplementary Table S2. The relationship between degradation ratio of histidine and *RSA*, p*K*a and *k*_2_ values of individual histidine residues in mAb drugs. 17

Supplementary Table S3. The amino acid sequences of adalimumab and rituximab. 18


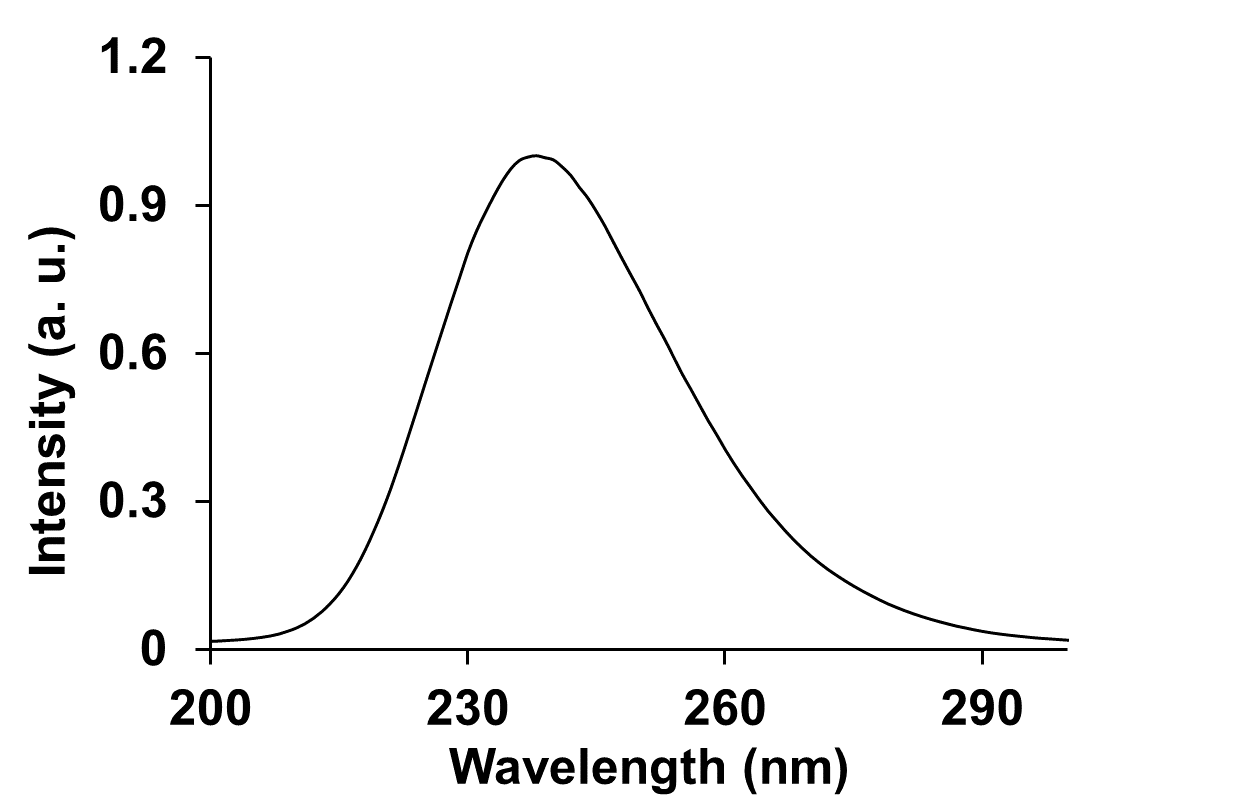


Supplementary Figure S1

Emission spectrum of the UVC light source PDUVL.


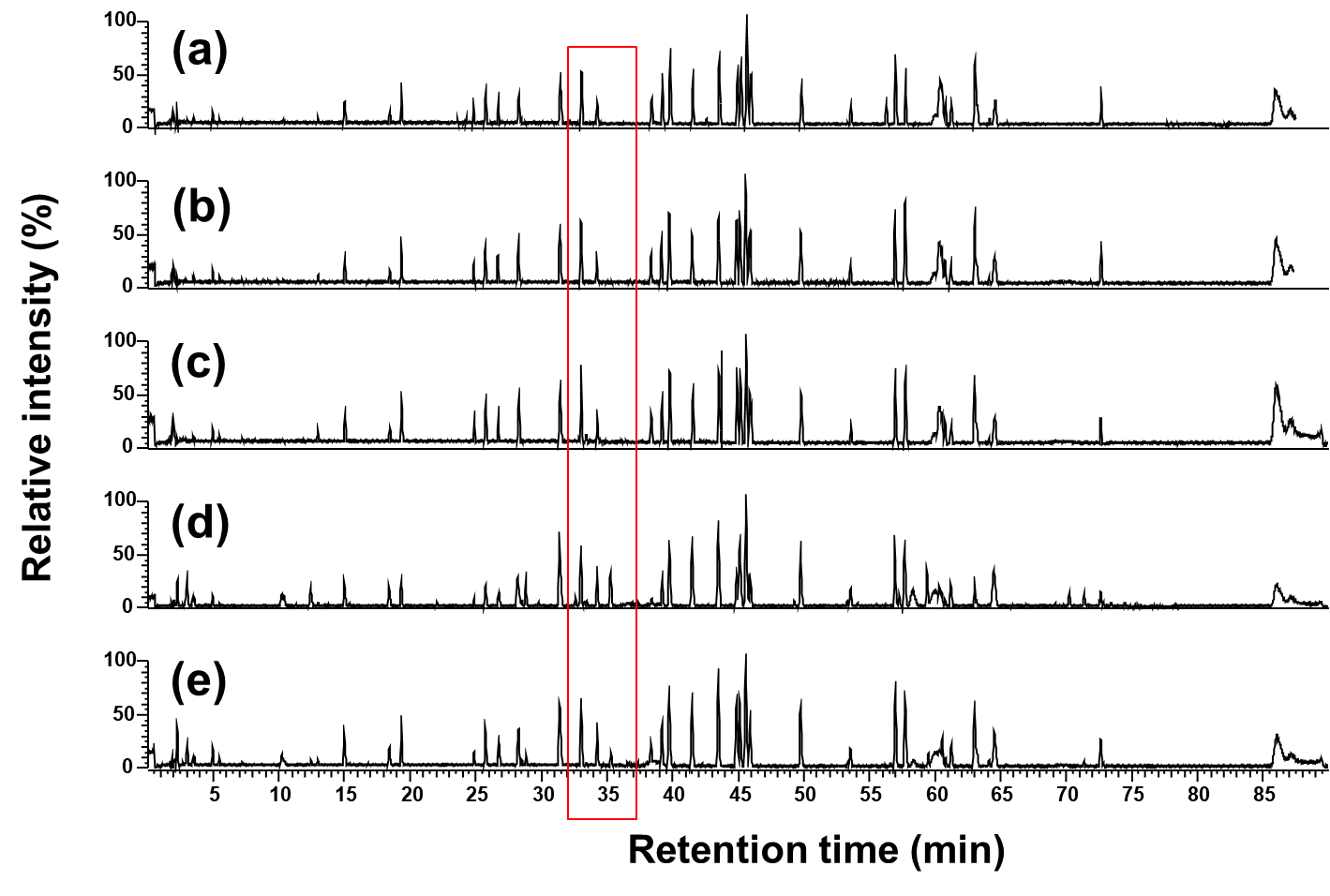


Supplementary Figure S2

Base peak ion chromatograms of peptides in the tryptic digests of adalimumab. (a) initial and adalimumab after UVC irradiation for (b) 1 min, (c) 5 min, (d) 15 min, and (e) 30min. The enlarged chromatograms enclosed in red rectangle in the range of 32 – 38 min are shown in Fig. 1.


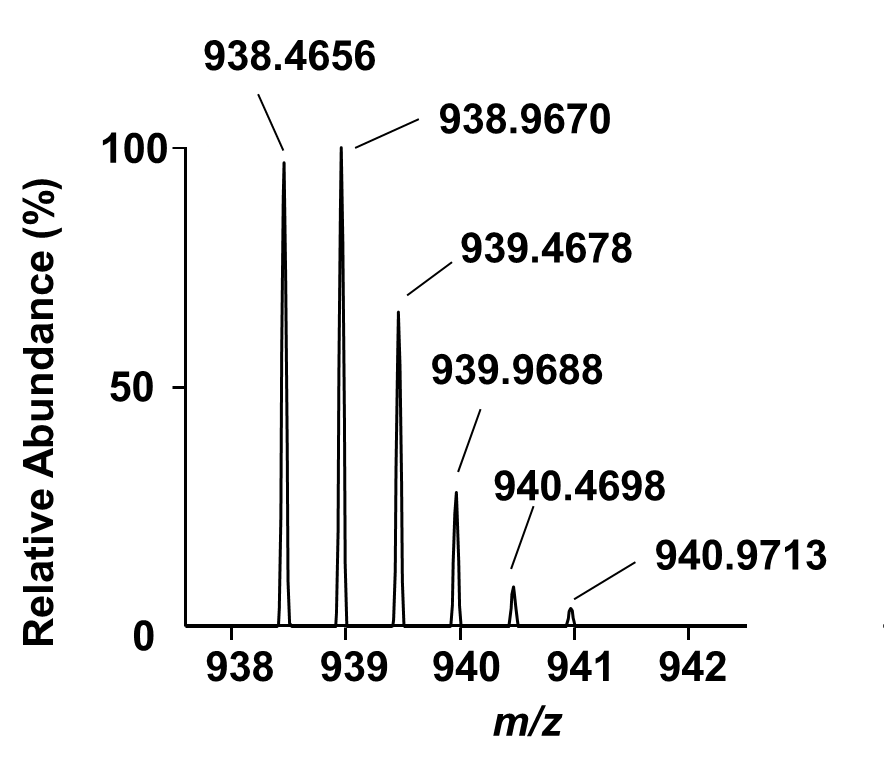


Supplementary Figure S3

Mass spectrum of the peptide that appeared as a peak at 33.0 min (Fig.1a). The most abundant isotopic peak is *m/z* 938.9678, and the monoisotopic peak is *m/z* 938.4656.


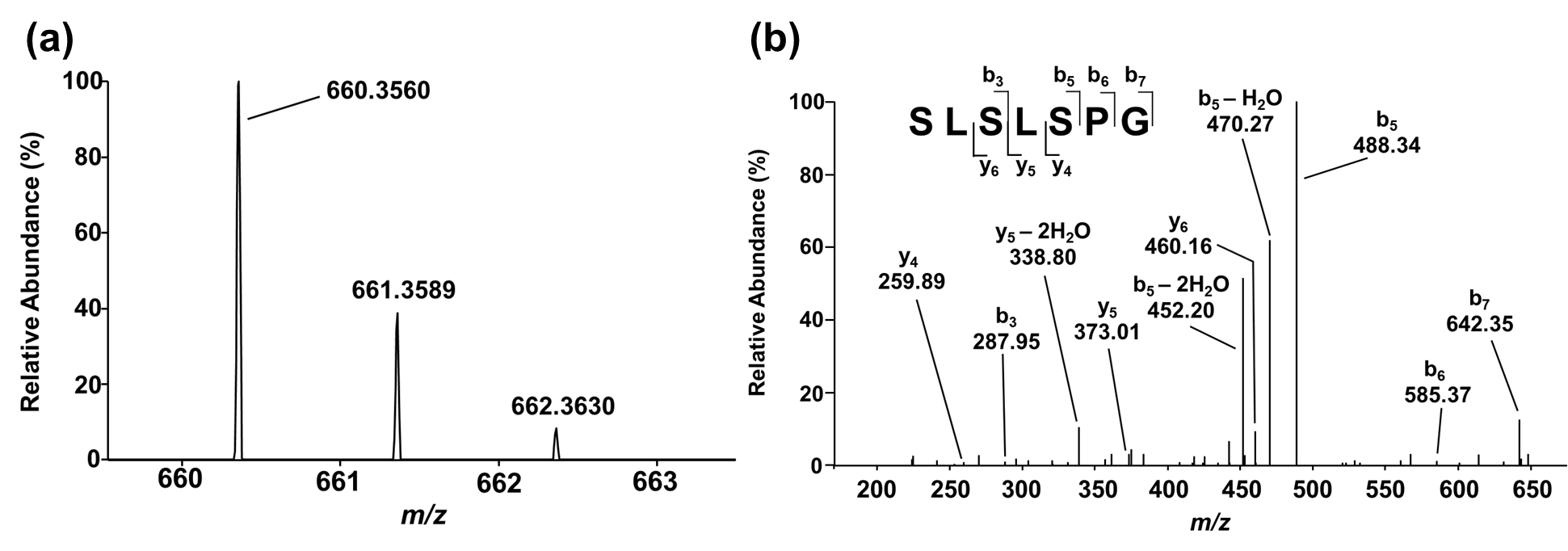


Supplementary Figure S4

(a) Mass spectrum of the peptide that appeared as a peak at 34.2 min (Fig. 1a). (b) MS/MS spectrum of the precursor peak at *m/z* 660.3560 in (a).


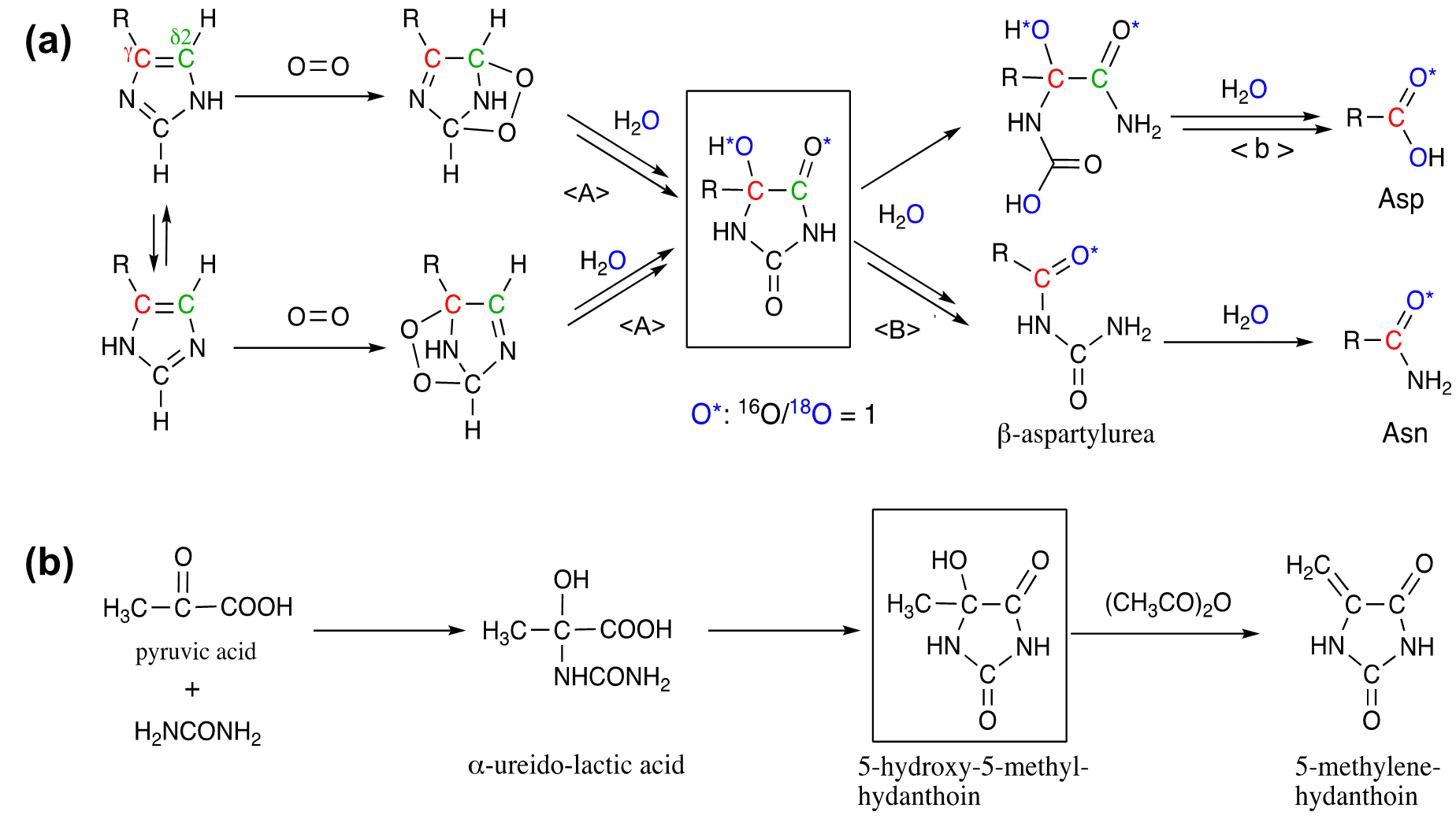


Supplementary Figure S5

(a) A mechanism of sensitized photooxidation of His as proposed by Tomita *et al*.^4^ The initial step of reaction between His and singlet oxygen is assumed to proceed in the manner of symmetry-allowed [4 + 2] cycloaddition. We modified Scheme 1 of Ref. 4 so that the incorporation of ^18^O from H_2_^18^O into the products can be discerned easily. Only a few intermediates of note including 5-alkyl-5-hydroxy-hydantoin (enclosed in rectangles) are shown. According to this mechanism, the ^18^O atoms in H_2_^18^O can be incorporated into both Asp (1.5 atoms) and Asn (0.5 atoms). However, our experimental results did not support this mechanism but made us to elaborate an alternative one as illustrated in Scheme 1 and Scheme S2. The mechanisms of hydration <A> and the cleavage of C^γ^−C^δ2^ bonds <B> still remain ambiguous. (b) The synthesis and reaction of 5-alkyl-5-hydroxy-hydantoin, which has been isolated as a stable compound and characterized (Murahashi, S.; Yuki, H.; Kosai, K.; Doura, F. Methylene-hydanthoin and related compounds. I. On the reaction of pyruvic acid and urea: the synthesis of 5-methylene-hydantoin. *Bull. Chem. Soc. Jpn*. 1966, *39*, 1559-1562). This suggests that the degradation step <B> does not proceed spontaneously, even if this intermediate should be formed in the preceding step <A>.


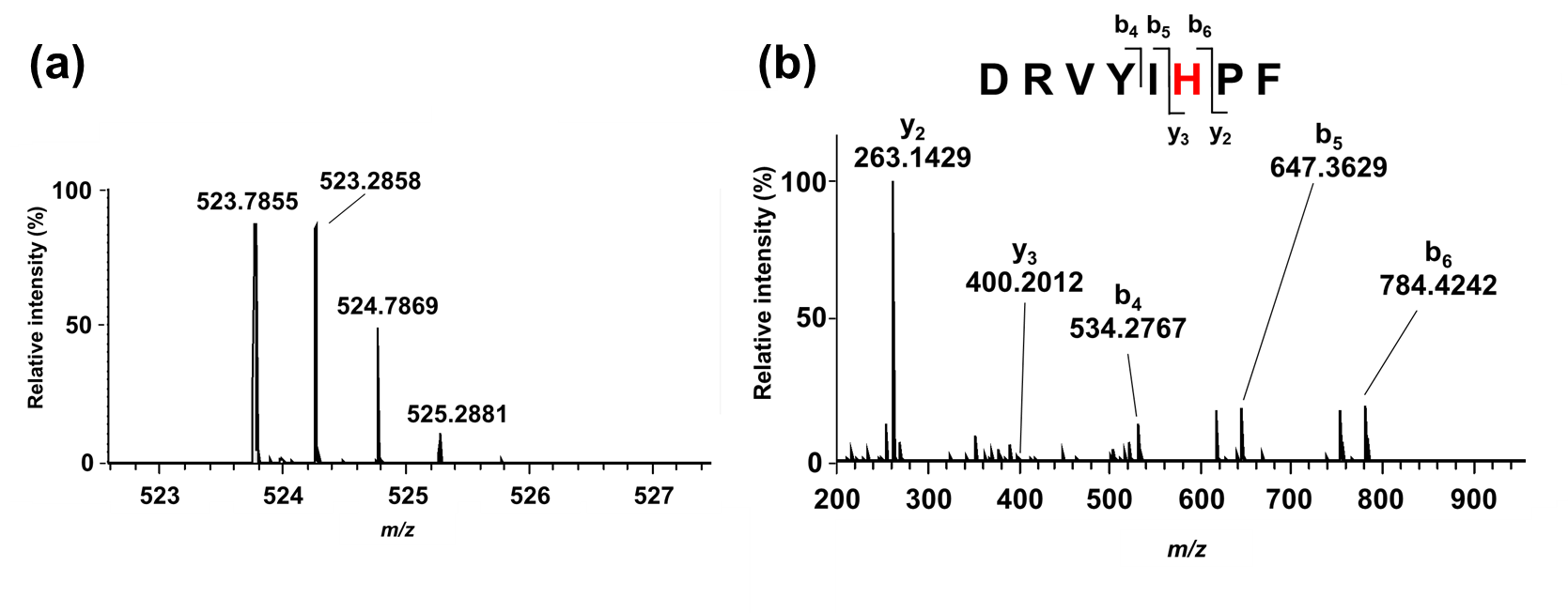


**Supplementary Figure S6**

(a) Mass spectrum of the peptide that appeared as a peak at 8.3 min (Fig. 3a). (b) MS/MS spectrum of the precursor peak at *m/z* 523.7855 in (a).


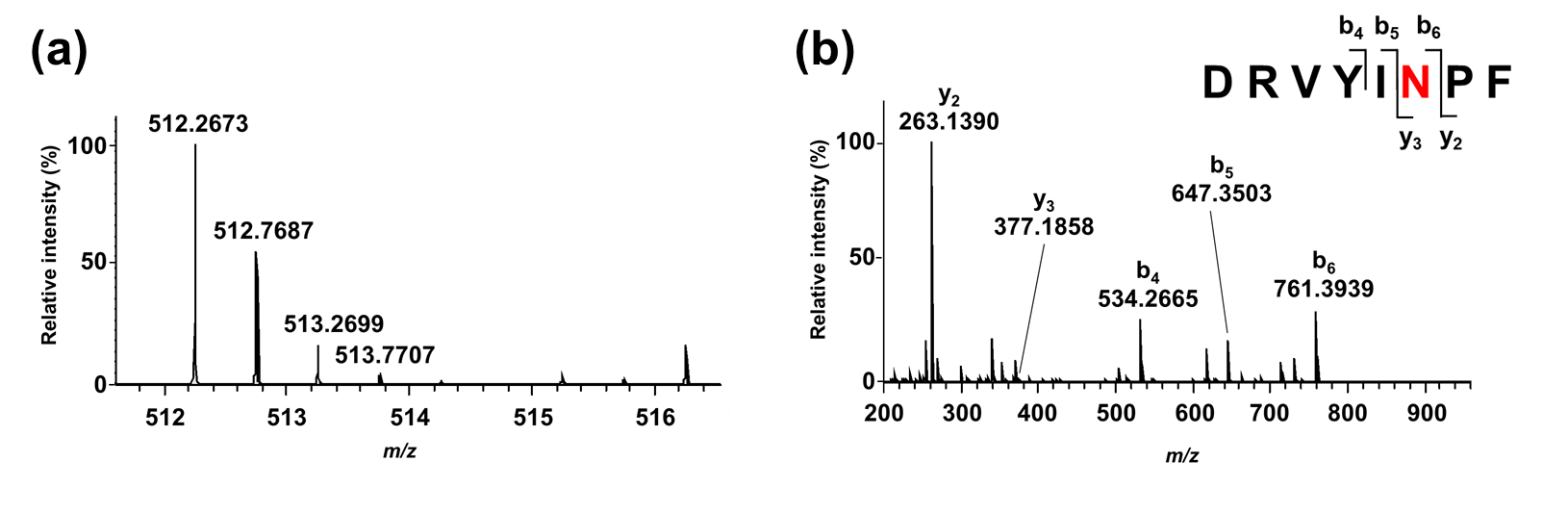


Supplementary Figure S7

(a) Mass spectrum of the photo-oxidation product *P*_4_ that appeared as a peak at 9.2 min (Figure 3b). (b) MS/MS spectrum of the precursor peak at *m/z* 512.2673 in spectrum (a). The spectra were taken for the peak of *P*_4_ appeared in the H_2_^16^O solution of angiotensin II after UVC irradiation for 60 min. The spectra (a) and (b) are indistinguishable from those shown as Figures 4a and 4b, respectively, obtained for the peak *P*_4_*'* that appeared in the H_2_^18^O solution.


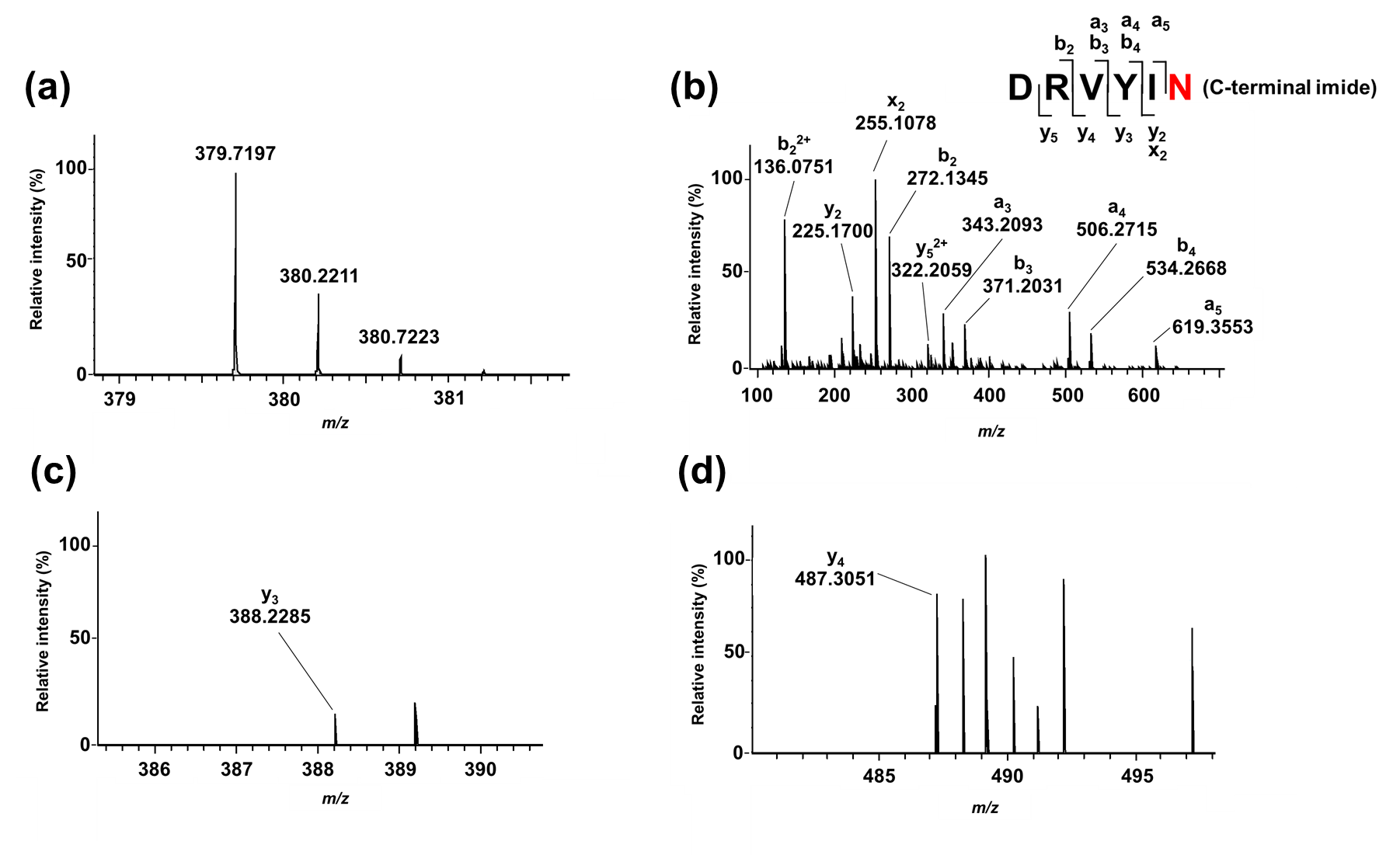


Supplementary Figure S8

(a) Mass spectrum of the photo-oxidation product *P*_1_ that appeared as a peak at 6.7 min (Fig. 3b). (b) MS/MS spectrum of the precursor peak at *m/z* 379.7197 in (a). Enlargements of the regions *m/z* 386 – 390 (c) and *m/z* 485 – 495 (d) in spectrum (b). We identified this product as DRVYIN (C-terminal imide), with a mass of 760.39 Da. For the structure and the possible mechanism of formation of this compound, see Supplementary Fig. S9.

**Supplementary Figure S9a**

A possible mechanism for the cleavage of the Asn-Pro bond to form the C-terminal Asn-imide. It also includes iso-Asp formation (d) through a transpeptidation. These reactions could occur individually from two isomeric forms of an intermediate (top), which is assumed to occur after the consecutive reactions of [2 + 2] cycloaddition and elimination (Fig. 5). This mechanism is consistent with the results of the UVC-induced photo-oxidation of His performed in H_2_^18^O, from which ^18^O atoms were incorporated only into Asp ([^18^O]Asp) but not into Asn. Although we illustrate here the mechanism of acid-catalyzed reactions of the intermediate in **Ia** form of Fig. 5, it is also possible to explain that of the base-catalyzed reactions in a similar manner or starting from intermediate in **Ib** form. Note that the intermediate is in an equilibrium mixture of tautomeric isomers **Ia** and **Ib**, which are interchangeable through the migration of proton.


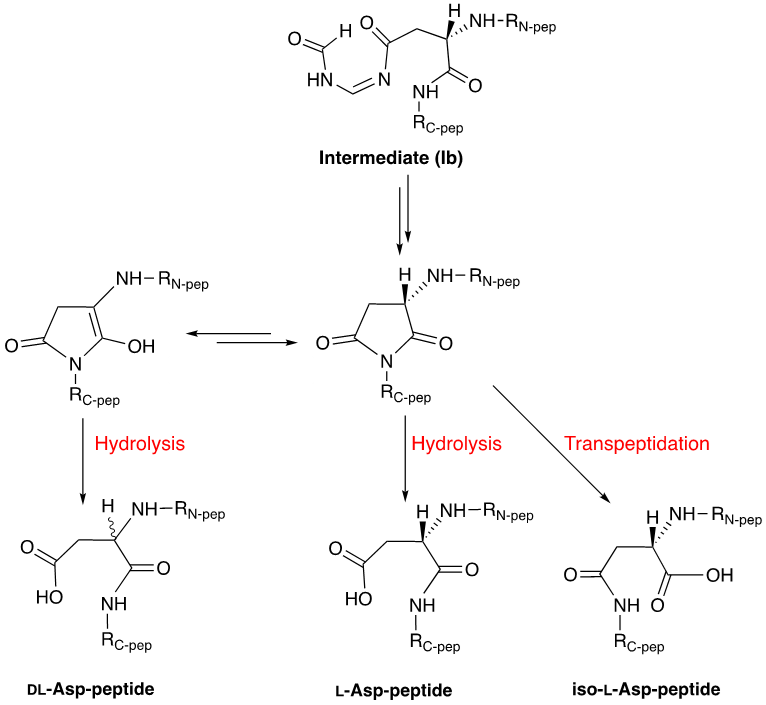


**Supplementary Figure S9b**

An extended mechanism for the formation of oxidation products including the racemic mixture of Asp residues. Note that all of these products can be derived from intermediate **Ib** and the subsequent five-membered succinimide as common precursors. It is widely accepted that the succinimide is racemization prone. (McFadden, P. N., Clarke, S. (1982) Methylation at d-aspartyl residues in erythrocytes: possible step in the repair of aged membrane proteins. *Proc Natl Acad Sci USA* **79**, 2460–2464.)


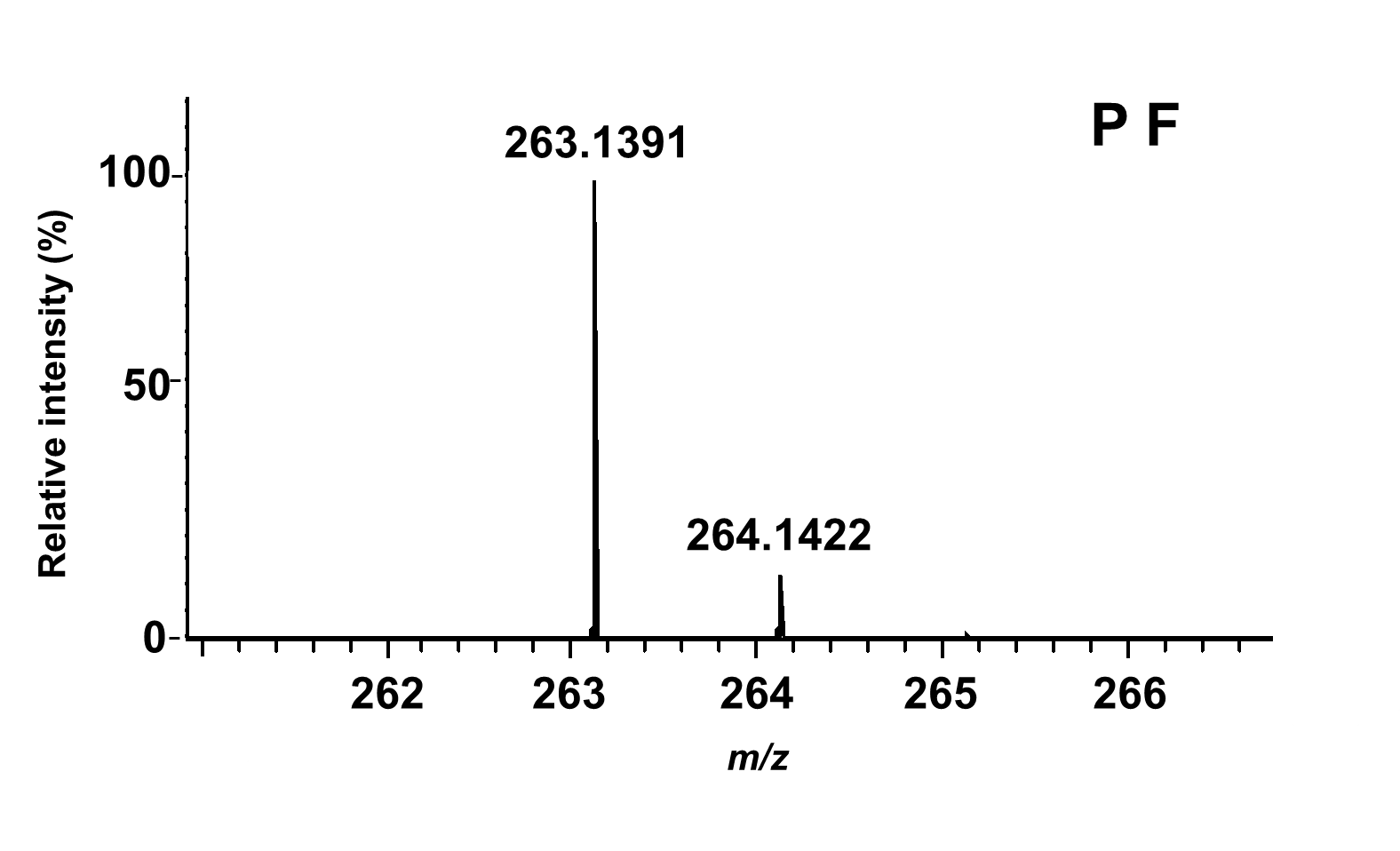


Supplementary Figure S10

Mass spectrum of the photo-oxidation product *P*_2_ that appeared as a peak at 7.5 min (Fig. 3b). We identified this product as a peptide PF (262.14 Da) corresponding to the C-terminal dipeptide of angiotensin II (DRVYINPF). For the mechanism of the formation of this peptide, see Supplementary Fig. S9.


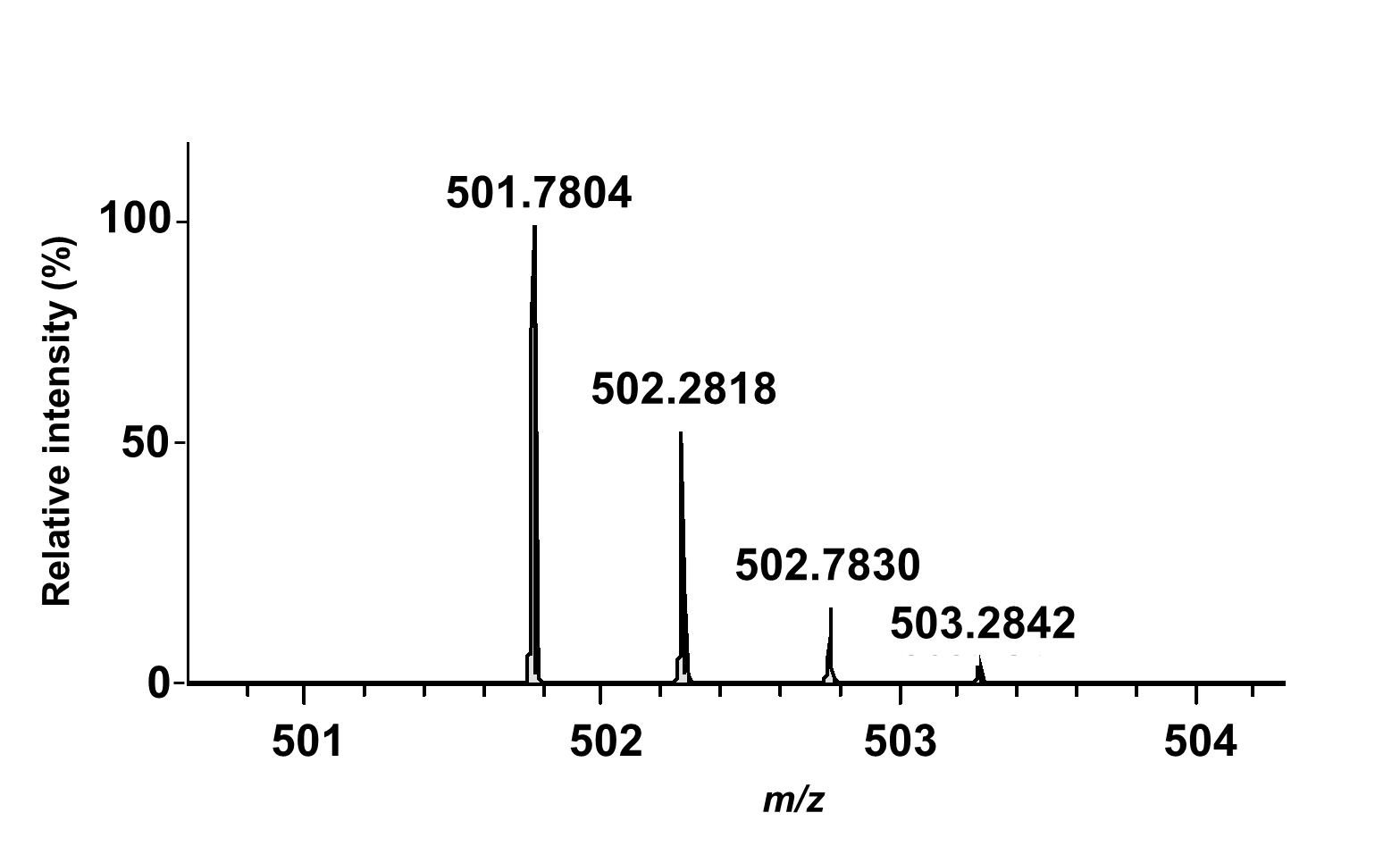


Supplementary Figure S11

Mass spectrum of the photo-oxidation product *P*_3_ that appeared as a minor peak at 8.8 min (Fig. 3a). The characterization of this compound is now in progress.


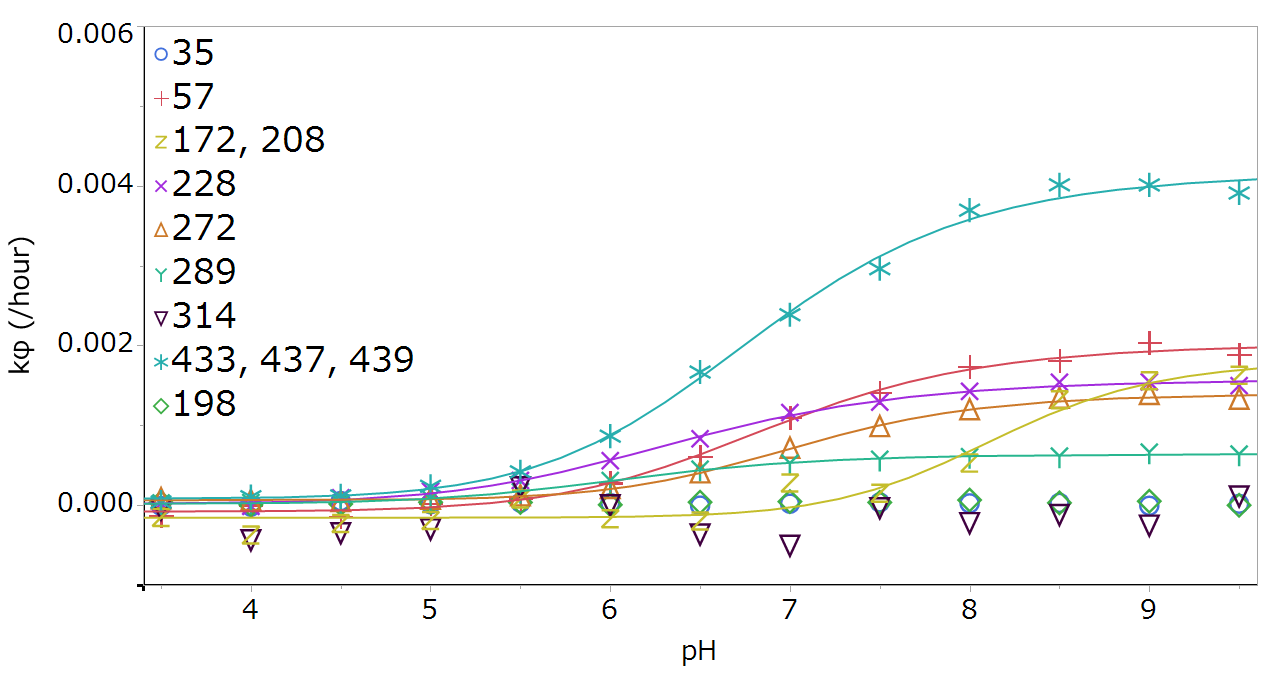


**Supplementary Figure S12**

pH dependence of pseudo-first-order rate constants for H/ D exchange at the C2-position of the histidine residues.


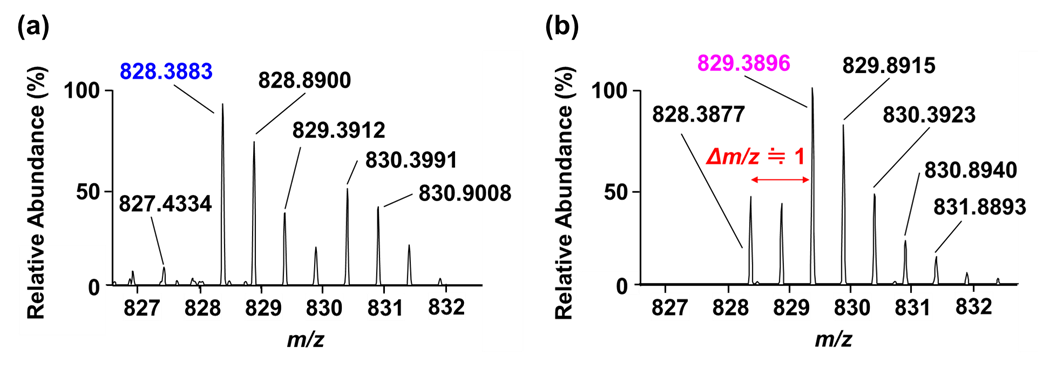


**Supplementary Figure S13**

Mass spectrum of the peptide was identified as residue 279-292 of adalimumab containing His289 in the amino acid sequence of FNWYVDGVEV(H oxidized to D)NAK (calculated monoisotopic mass of 1654.76 Da). The spectrum were taken for the solutions of adalimumab after UVC irradiation for 30 min in H_2_^16^O (a) and in H_2_^18^O (b). We confirmed the incorporation of ^18^O into Asp from the solvent water.

| **His-Peptide** | | | | | | **Product Peptide ^c^** | | | | **Δ*M*_H/X_/Da ^d^ Product (N/D)** | **Yield ^g^  (%)** |
| --- | --- | --- | --- | --- | --- | --- | --- | --- | --- | --- | --- |
| **Retention time  (min)** | **Base peak *m/z* ^a^** | **Monoisotopic**  **mass (Da)** | ***M*_H_ /Da (calculated)** | **Peptide ^b^** | **Residue**  **No.**  **of His** | **Retention time  (min)** | **Base peak *m/z* ^a^** | **Monoisotopic**  **mass (Da)** | ***M*_X_ /Da (calculated)** |  |  |
| **58.4** | **834.4145** | **3333.6267** | **3333.6348** | **S223-K252 (ADL, HC)** | **228** | **60.7** | **828.6597** | **3310.6075** | **3310.6189** | **23.0192 (N)** | **N.D. ^f^** |
| **60.3** | **948.8233** | **2843.4464** | **2843.4502** | **T227-K252 (RTX, HC)** | **228** | **63.7** | **941.1498** | **2820.4259** | **2820.4343** | **23.0205 (N)** | **N.D. ^f^** |
|  |  |  |  |  |  | **64.2** | **941.4786** | **2821.4123** | **2821.4183** | **22.0341 (D)** | **N.D. ^f^** |
| **45.5** | **1070.0154** | **2138.0151** | **2138.0201** | **T260-K278 (ADL, HC)** | **272** | **47.0** | **1058.5073** | **2114.9989** | **2115.0041** | **23.0162 (N)** | **1.4** |
|  |  |  |  |  |  | **47.6** | **1058.9989** | **2115.9821** | **2115.9882** | **22.0330 (D)** | **3.0** |
| **45.6** | **1070.0151** | **2138.0145** | **2138.0201** | **T260-K278 (RTX, HC)** | **272** | **47.0** | **1058.5066** | **2114.9975** | **2115.0041** | **23.0170 (N)** | **1.7** |
|  |  |  |  |  |  | **47.6** | **1058.9989** | **2115.9821** | **2115.9882** | **22.0324 (D)** | **3.9** |
| **45.1** | **839.4034** | **1676.7911** | **1676.7947** | **F279-K292 (ADL, HC)** | **289** | **49.0** | **827.8950** | **1653.7743** | **1653.7787** | **23.0168 (N)** | **3.6** |
|  |  |  |  |  |  | **50.0** | **828.3879** | **1654.7601** | **1654.7627** | **22.0310 (D)** | **2.6** |
| **45.1** | **839.4033** | **1676.7909** | **1676.7947** | **F279-K292 (RTX, HC)** | **289** | **49.1** | **827.8950** | **1653.7743** | **1653.7787** | **23.0166 (N)** | **3.3** |
|  |  |  |  |  |  | **50.0** | **828.3877** | **1654.7597** | **1654.7627** | **22.0312 (D)** | **7.2** |
| **59.5** | **743.4067** | **2227.1966** | **2227.2001** | **V306-K324 (ADL, HC)** | **314** | **65.4** | **736.0624** | **2205.1637** | **2205.1681** | **22.0329 (D)** | **N.D. ^f^** |
| **62.9** | **904.5079** | **1807.0001** | **1806.9993** | **V306-K321 (RTX, HC)** | **314** | **69.4** | **892.9980** | **1783.9803** | **1783.9857** | **23.0198 (N)** | **4.5** |
|  |  |  |  |  |  | **72.0** | **893.4895** | **1784.9639** | **1784.9645** | **22.0362 (D)** | **4.9** |
| **43.4** | **701.0718** | **2800.2559** | **2800.2598** | **W421-K443 ^e^ (ADL, HC)** | **437 ^e^** | **46.1** | **695.5640** | **2778.2239** | **2778.2279** | **22.0320 (D)** | **1.7** |
|  |  |  |  |  | **439 ^e^** | **46.9** | **695.3173** | **2777.2379** | **2777.2439** | **23.0180 (N)** | **1.3** |
|  |  |  |  |  |  | **47.9** | **695.5638** | **2778.2247** | **2778.2279** | **22.0312 (D)** | **2.3** |
| **43.4** | **701.0718** | **2800.2559** | **2800.2598** | **W421-K443 ^e^ (RTX, HC)** | **433 ^e^** | **46.9** | **695.5634** | **2778.2223** | **2778.2279** | **22.0336 (D)** | **2.7** |
|  |  |  |  |  | **437 ^e^** | **46.1** | **695.3176** | **2777.2391** | **2777.2439** | **23.0168 (N)** | **1.7** |
|  |  |  |  |  |  | **47.9** | **695.5635** | **2778.2227** | **2778.2279** | **22.0332 (D)** | **1.6** |
|  |  |  |  |  | **439 ^e^** | **48.5** | **695.3171** | **2777.2371** | **2777.2427** | **23.0188 (N)** | **N.D. ^f^** |
| **28.2** | **714.3649** | **2140.0712** | **2140.0735** | **H189-K207 ^f^ (ADL, LC)** | **189** | **32.6** | **706.6918** | **2117.0519** | **2117.0575** | **23.0193 (N)** | **N.D. ^f^** |
|  |  |  |  |  | **198** | **31.2** | **706.6920** | **2117.0525** | **2117.0575** | **23.0187 (N)** | **N.D. ^f^** |
|  |  |  |  |  |  | **32.6** | **707.0186** | **2118.0323** | **2118.0416** | **22.0389 (D)** | **N.D. ^f^** |
| **33.0** | **938.4656** | **1874.9155** | **1874.9196** | **V191-K207 (ADL, LC)** | **198** | **36.3** | **926.9572** | **1851.9041** | **1851.9037** | **23.0114 (N)** | **4.0** |
|  |  |  |  |  |  | **35.4** | **927.4487** | **1852.8821** | **1852.8877** | **22.0334 (isoD)** | **4.9 ^h^** |
|  |  |  |  |  |  | **37.6** | **927.4495** | **1852.8833** | **1852.8877** | **22.0322 (D)** |  |
| **33.0** | **938.4665** | **1874.9173** | **1874.9196** | **V190-K206 (RTX, LC)** | **197** | **36.3** | **926.9579** | **1851.9001** | **1851.9037** | **23.0172 (N)** | **4.4** |
|  |  |  |  |  |  | **35.4** | **927.4493** | **1852.8839** | **1852.8877** | **22.0334 (isoD)** | **8.2 ^h^** |
|  |  |  |  |  |  | **37.6** | **927.4498** | **1852.8829** | **1852.8877** | **22.0344 (D)** |  |

**Supplementary Table S1**

Degradation ratio of individual histidine residues in mAb drugs after UVC irradiation for 30 min.

^a^ Allowed for the error of *m/z* ± 10 ppm to extract the peak to evaluate the degradation ratio in mass chromatograms.

^b^ Full amino acid sequences of adalimumab (ADL) and rituximab (RTX) with heavy chains (HC) and light chains (LC) are given in Supplementary Table S3.

^c^ Losses of mass by 23 Da for Asn and by 22 Da for Asp are associated with the degradation ratio of His.

^d^ The difference in molecular masses *M*_H_ – *M*_X_ (X = Asp or Asn) of peptides associated with the modification of His to either Asp or Asn.

^e^ There are three His residues in the peptide W421−K443. Their degradation ratios were calculated individually.

^f^ N.D.: Not determined. It was difficult to quantify the degradation ratio due to the very low peak intensities.

^g^ We referred to yield as the ratio of peak area of Asn or Asp to the total area of all the peaks arising from a given His residue.

^h^ Calculated by including an isomer of aspartic acid (*e.g*., Asp or iso-Asp).

| Chain | Residue  number  of His | Degradation ratio of His at UVC irradiation 30 min (%) | | *RSA* (%) | | p*K*_a_ | His C_2_-H/D exchange reaction | |
| --- | --- | --- | --- | --- | --- | --- | --- | --- |
|  |  |  |  |  |  |  | *t*_1/2_  (day) | *k*_2_  (×10^4^∙M^-1^∙h^-1^) |
|  |  | Adalimumab | Rituximab | Adalimumab | Rituximab |  |  |  |
| Heavy  chain | 35 | N.D. ^a^ | N.D. | 7 | 5 | N.D. | 45 > | N.D. |
|  | 57 ^b^ | N.D. |  | 41 |  | 6.9 | 14 | 2.5 |
|  | 172 | N.D. | N.D. | 6 | 6 | 8.1 ^f^ | 16 ^f^ | 0.1^f^ |
|  | 208 | N.D. | N.D. | 0 | 0 |  |  |  |
|  | 228 | N.D. | N.D. | 19 | 30 | 6.4 | 18 | 6.3 |
|  | 272 | 4.4 | 5.6 | 21 | 21 | 7.0 | 21 | 1.4 |
|  | 289 | 6.2 | 10.5 | 48 | 48 | 6.2 | 45 | 4.1 |
|  | 314 | N.D. | 9.4 | 13 | 13 | N.D. | 45 > | N.D. |
|  | 433 | N.D. | 2.7 | 0 | 0 | 6.8 ^g^ | 7.0 ^g^ | 6.6 ^g^ |
|  | 437 | 1.7 | 3.3 | 53 | 53 |  |  |  |
|  | 439 | 3.6 | N.D. | 21 | 21 |  |  |  |
| Light  chain | 33 ^c^ |  | N.D. |  | 1 | N.D. | N.D. | N.D. |
|  | 189 ^d^ | N.D. | N.D. | 14 | 14 | N.D. | N.D. | N.D. |
|  | 198 ^e^ | 8.9 | 12.6 | 5 | 2 | N.D. | 45 > | N.D. |

**Supplementary Table S2**

The relationship between degradation ratio of histidine and *RSA*, p*K*_a_ and *k*_2_ values of individual histidine residues in mAb drugs.

^a^ N.D.: Not detected (or not determined).

^b^ Not observed in rituximab.

^c^ Not observed in adalimumab.

^d^ His188 in rituximab.

^e^ His197 in rituximab.

^f^ Average of the two histidine residues.

^g^ Average of the three histidine residues.

| Adalimumab | Heavy  chain | EVQLVESGGG LVQPGRSLRL SCAASGFTFD DYAM**H**WVRQA PGKGLEWVSA ITWNSG**H**IDY ADSVEGRFTI SRDNAKNSLY LQMNSLRAED TAVYYCAKVS YLSTASSLDY WGQGTLVTVS SASTKGPSVF PLAPSSKSTS GGTAALGCLV KDYFPEPVTV SWNSGALTSG V**H**TFPAVLQS SGLYSLSSVV TVPSSSLGTQ TYICNVN**H**KP SNTKVDKKVE PKSCDKT**H**TC PPCPAPELLG GPSVFLFPPK PKDTLMISRT PEVTCVVVDV S**H**EDPEVKFN WYVDGVEV**H**N AKTKPREEQY NSTYRVVSVL TVL**H**QDWLNG KEYKCKVSNK ALPAPIEKTI SKAKGQPREP QVYTLPPSRD ELTKNQVSLT CLVKGFYPSD IAVEWESNGQ PENNYKTTPP VLDSDGSFFL YSKLTVDKSR WQQGNVFSCS VM**H**EAL**H**N**H**Y TQKSLSLSPG K |
| --- | --- | --- |
|  | Light  chain | DIQMTQSPSS LSASVGDRVT ITCRASQGIR NYLAWYQQKP GKAPKLLIYA ASTLQSGVPS RFSGSGSGTD FTLTISSLQP EDVATYYCQR YNRAPYTFGQ GTKVEIKRTV AAPSVFIFPP SDEQLKSGTA SVVCLLNNFY PREAKVQWKV DNALQSGNSQ ESVTEQDSKD STYSLSSTLT LSKADYEK**H**K VYACEVT**H**QG LSSPVTKSFN RGEC |
| Rituximab | Heavy  chain | QVQLQQPGAE LVKPGASVKM SCKASGYTFT SYNM**H**WVKQT PGRGLEWIGA IYPGNGDTSY NQKFKGKATL TADKSSSTAY MQLSSLTSED SAVYYCARST YYGGDWYFNV WGAGTTVTVS AASTKGPSVF PLAPSSKSTS GGTAALGCLV KDYFPEPVTV SWNSGALTSG V**H**TFPAVLQS SGLYSLSSVV TVPSSSLGTQ TYICNVN**H**KP SNTKVDKKAE PKSCDKT**H**TC PPCPAPELLG GPSVFLFPPK PKDTLMISRT PEVTCVVVDV S**H**EDPEVKFN WYVDGVEV**H**N AKTKPREEQY NSTYRVVSVL TVL**H**QDWLNG KEYKCKVSNK ALPAPIEKTI SKAKGQPREP QVYTLPPSRD ELTKNQVSLT CLVKGFYPSD IAVEWESNGQ PENNYKTTPP VLDSDGSFFL YSKLTVDKSR WQQGNVFSCS VM**H**EAL**H**N**H**Y TQKSLSLSPG K |
|  | Light  chain | QIVLSQSPAI LSASPGEKVT MTCRASSSVS YI**H**WFQQKPG SSPKPWIYAT SNLASGVPVR FSGSGSGTSY SLTISRVEAE DAATYYCQQW TSNPPTFGGG TKLEIKRTVA APSVFIFPPS DEQLKSGTAS VVCLLNNFYP REAKVQWKVD NALQSGNSQE SVTEQDSKDS TYSLSSTLTL SKADYEK**H**KV YACEVT**H**QGL SSPVTKSFNR GEC |

Supplementary Table S3

The amino acid sequences of adalimumab and rituximab. His residues are printed in bold blue. The amino acid sequences of peptides containing His residue(s) identified in the present study are highlighted in yellow (adalimumab) and in light blue (rituximab). The C-terminal peptide (SLSLSPG) of adalimumab heavy chain is colored in green.
